# Unraveling the genetic variations underlying virulence disparities among SARS-CoV-2 strains across global regions: insights from Pakistan

**DOI:** 10.1186/s12985-024-02328-8

**Published:** 2024-03-06

**Authors:** Momina Jabeen, Shifa Shoukat, Huma Shireen, Yiming Bao, Abbas Khan, Amir Ali Abbasi

**Affiliations:** 1 National Center for Bioinformatics, Program of Comparative and Evolutionary Genomics, Faculty of Biological Sciences, Quaid-i-Azam University, 45320 Islamabad, Pakistan; 2 National Genomics Data Center & CAS Key Laboratory of Genome Sciences and Information, Beijing Institute of Genomics, Chinese Academy of Sciences, China National Center for Bioinformation, 100101 Beijing, China; 3 University of Chinese Academy of Sciences, 100101 Beijing, China; 4 Department of Bioinformatics and Biological Statistics, School of Life Sciences and Biotechnology, Shanghai Jiao Tong University, 200240 Shanghai, China; 5 School of Medical and Life Sciences, Sunway University, Sunway City, Malaysia

**Keywords:** SARS-CoV-2, COVID-19, Macrodomains, pp1ab polyprotein, Nsp3, Viral fitness

## Abstract

Over the course of the COVID-19 pandemic, several SARS-CoV-2 variants have emerged that may exhibit different etiological effects such as enhanced transmissibility and infectivity. However, genetic variations that reduce virulence and deteriorate viral fitness have not yet been thoroughly investigated. The present study sought to evaluate the effects of viral genetic makeup on COVID-19 epidemiology in Pakistan, where the infectivity and mortality rate was comparatively lower than other countries during the first pandemic wave. For this purpose, we focused on the comparative analyses of 7096 amino-acid long polyprotein pp1ab. Comparative sequence analysis of 203 SARS-CoV-2 genomes, sampled from Pakistan during the first wave of the pandemic revealed 179 amino acid substitutions in pp1ab. Within this set, 38 substitutions were identified within the Nsp3 region of the pp1ab polyprotein. Structural and biophysical analysis of proteins revealed that amino acid variations within Nsp3’s macrodomains induced conformational changes and modified protein-ligand interactions, consequently diminishing the virulence and fitness of SARS-CoV-2. Additionally, the epistatic effects resulting from evolutionary substitutions in SARS-CoV-2 proteins may have unnoticed implications for reducing disease burden. In light of these findings, further characterization of such deleterious SARS-CoV-2 mutations will not only aid in identifying potential therapeutic targets but will also provide a roadmap for maintaining vigilance against the genetic variability of diverse SARS-CoV-2 strains circulating globally. Furthermore, these insights empower us to more effectively manage and respond to potential viral-based pandemic outbreaks of a similar nature in the future.

**Supplementary Information:**

The online version contains supplementary material available at 10.1186/s12985-024-02328-8.

## Background

Severe acute respiratory syndrome coronavirus 2 (SARS-CoV-2), the causative agent of coronavirus disease 2019 (COVID-19), initially emerged in Wuhan, China, in December 2019 [1]. Since its emergence, the virus has rapidly propagated through human-to-human transmission, giving rise to a significant global public health challenge [2]. The widespread dispersion of the virus prompted the World Health Organization (WHO) to designate the situation as a pandemic on March 11, 2020 [3]. As of February 2024, the global tally indicates approximately 702 million confirmed infections and around 6.97 million reported deaths (https://www.worldometers.info/coronavirus/). Within this context, Pakistan, ranking as the sixth most populous country worldwide, with a population of roughly 233 million, has registered 1.58 million confirmed COVID-19 cases and recorded approximately 30,664 fatalities as of February 2024 (https://www.worldometers.info/coronavirus/country/pakistan/).

SARS-CoV-2, a beta coronavirus belonging to the Coronaviridae family, is evolutionarily related to bat coronaviruses, exhibiting approximately 96% sequence similarity with bat-CoV, specifically bat-RaTG13 [4]. The genome of SARS-CoV-2 comprises 29.8 kilobases (kb) of single-stranded positive-sense RNA (+ ssRNA). Positioned at the 5’-terminal portion are the two largest open reading frames (ORFs), namely ORF1a and ORF1ab, constituting two-thirds of the entire SARS-CoV-2 genome [5]. These ORFs give rise to two substantial polyproteins, pp1ab (7096 amino acids) and pp1a (4405 amino acids), which undergo proteolytic cleavage to yield 16 non-structural proteins (Nsps). These Nsps play a pivotal role in viral replication and immune evasion [6, 7]. Situated in the 3’-terminal region of the SARS-CoV-2 genome are the encoding sequences for four structural proteins: spike protein (S), membrane protein (M), envelope protein (E), and nucleocapsid protein (N), in addition to several accessory proteins [7].

Ever since the emergence of SARS-CoV-2, its genome has exhibited a susceptibility to genetic variations [8], with an approximate nucleotide substitution rate of 1 × 10^− 3^ per year [9]. These genetic changes have given rise to novel SARS-CoV-2 variants, characterized by substantial disparities in viral infectivity, transmissibility, and pathogenesis [8, 10]. Consequently, numerous studies have been conducted to investigate genomic variations, with a specific emphasis on the spike protein, which greatly facilitates the virus’s transmission [10]. For instance, the D614G mutation in the spike protein has been linked to an increase in SARS-CoV-2 transmissibility and virulence [11]. Beyond the spike protein, the virus encompasses numerous other constituents that contribute to its virulence, pathogenesis, and transmission. These include ORF3a, ORF1ab, ORF8, ORF10, N, M, E, ORF7, and ORF6 [10, 12, 13].

Given its high contagion and pathogenicity, it becomes imperative to delve into the structural components of SARS-CoV-2 that, under the influence of genetic variations, shape the host response and modify the virus’s pathogenicity, transmissibility, and infectivity across different geographic regions [12, 14, 15]. For instance, during the initial phase of the COVID-19 pandemic (February to October 2020), Iran and various European nations experienced elevated mortality rates, whereas Singapore, Malaysia, South Korea, and Pakistan witnessed a comparatively lower mortality rate [16–18]. Therefore, comprehending the genetic variations of SARS-CoV-2 across diverse human populations could offer insights into the underlying mechanisms behind the variability in its pathogenic impact. In alignment with this perspective, the present study was conceived to meticulously examine the genetic diversities present in all available pp1ab polyprotein sequences of SARS-CoV-2 isolated from the Pakistani populace during the inaugural wave of the pandemic (March 01, 2020, to June 30, 2020; encompassing a total of 203 complete genome sequences). Furthermore, our investigation centered on the substitutions of amino acids within the macrodomains of the non-structural protein 3 (Nsp3), aiming to anticipate the implications of their structural evolution on SARS-CoV-2’s virulence and pathogenesis.

## Methods

### Sequence acquisition

In this study, all available fully sequenced genomes of SARS-CoV-2 sampled from Pakistan during the initial wave of the COVID-19 pandemic, spanning from March 01, 2020, to June 30, 2020, were utilized. The majority of these genomes were obtained through large-scale genome sequencing initiatives [19]. To accomplish this, the polyprotein pp1ab sequences originating from the completely sequenced SARS-CoV-2 genomes were retrieved from various sources, including the National Center for Biotechnology Information (NCBI-virus) database [20], the Global Initiative on Sharing All Influenza Data (GISAID) [21], and the 2019 Novel Coronavirus Resource (2019-nCoVR) [22, 23]. In total, a collection of 203 polyprotein pp1ab sequences was amassed, each containing 7096 amino acid residues (Additional File 1: Table S1). Additionally, polyprotein pp1ab sequences hailing from the four genera within the subfamily Coronavirinae — Alphacoronavirus, Betacoronavirus, Gammacoronavirus, and Deltacoronavirus — were retrieved from the NCBI (Additional File 1: Table S2). Moreover, set of 10 sequences from each subsequent variants of SARS-CoV-2 i.e., alpha, beta, delta, and omicron were retrieved, that emerged in Pakistan after the first pandemic wave (Additional File 1: Table S9).

### Phylogenetic and sequence analysis

**Fig. 1:**
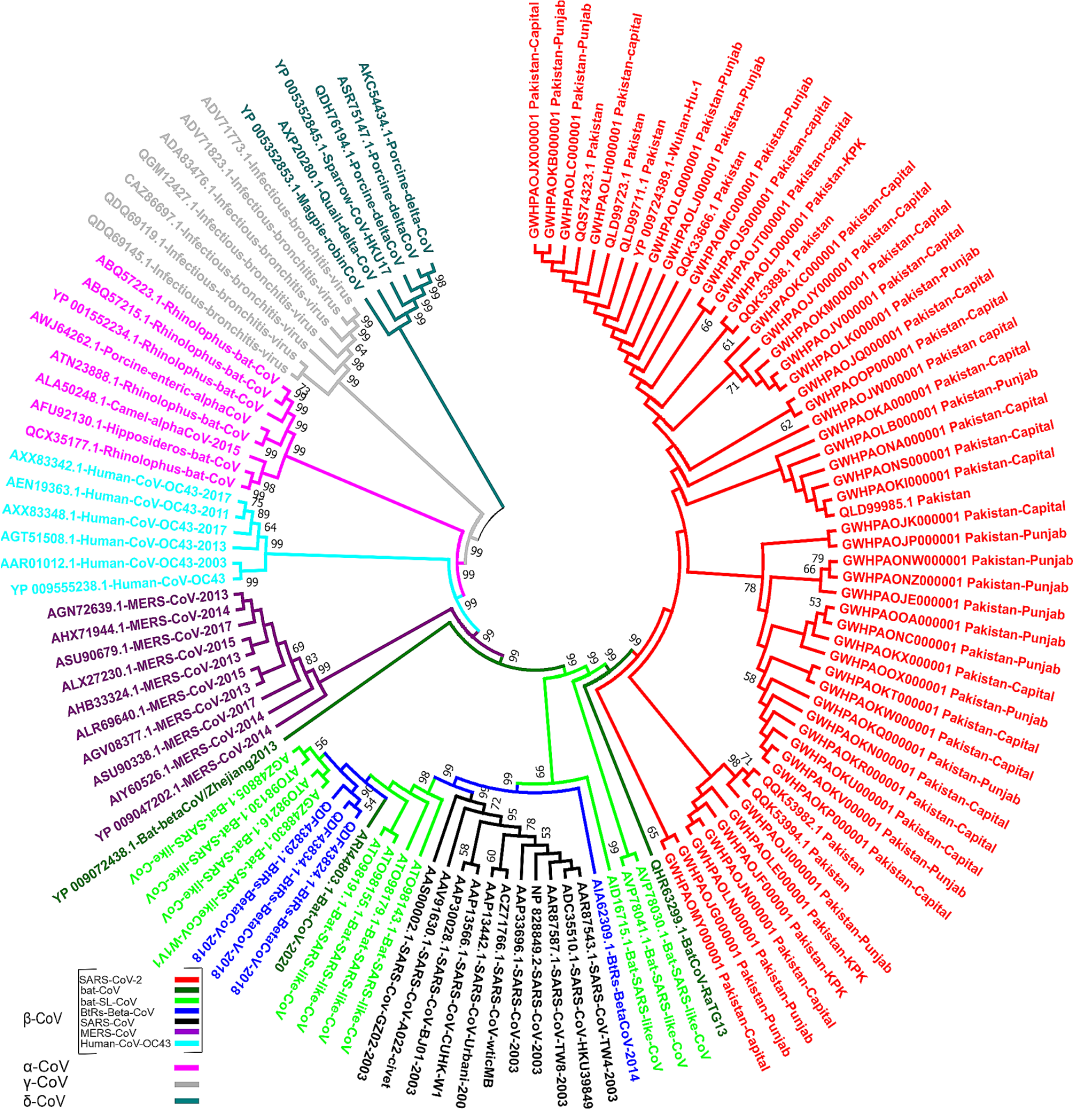
Phylogenetic characterization of SARS-CoV-2 based on 7096-aa polyprotein pp1ab: Phylogenetic tree demonstrating the relationship of SARS-CoV-2 strains isolated from Pakistan to other CoVs. Phylogenetic analysis involved 122 pp1ab sequences from the subfamily Coronavirinae, including representatives of four genera; α -CoV, β -CoV, γ -CoV, and δ -CoV (Note: 55 SARS-CoV-2 pp1ab sequences from Pakistan are included in phylogenetic analysis). The color codes distinguish between various groups/types of coronaviruses. The phylogenetic tree was reconstructed using the neighbor joining method with the p-distance as substitution model. Bootstrap values ≥ 50% are shown along the branches. A scaled phylogram of this tree with branch lengths reflecting the amount of genetic change is provided as Fig. S1 in Additional File 2

To initiate phylogenetic analysis, sequence alignment was performed on 122 Coronavirinae polyprotein pp1ab sequences, which included a representative subset of SARS-CoV-2 sequences from Pakistan. The alignment was carried out using the CLUSTALW tool (Additional File 1: Table S1, S2) [24]. Subsequently, the phylogenetic tree for the subfamily Coronavirinae was reconstructed using MEGA7, employing the neighbor-joining (NJ) method with the p-distance model for amino acid substitutions. To assess the robustness of the tree’s topology, bootstrap analysis with 1000 pseudoreplicates was conducted (Fig. 1; Additional File 2: Fig. S1) [25, 26].

**Fig. 2:**
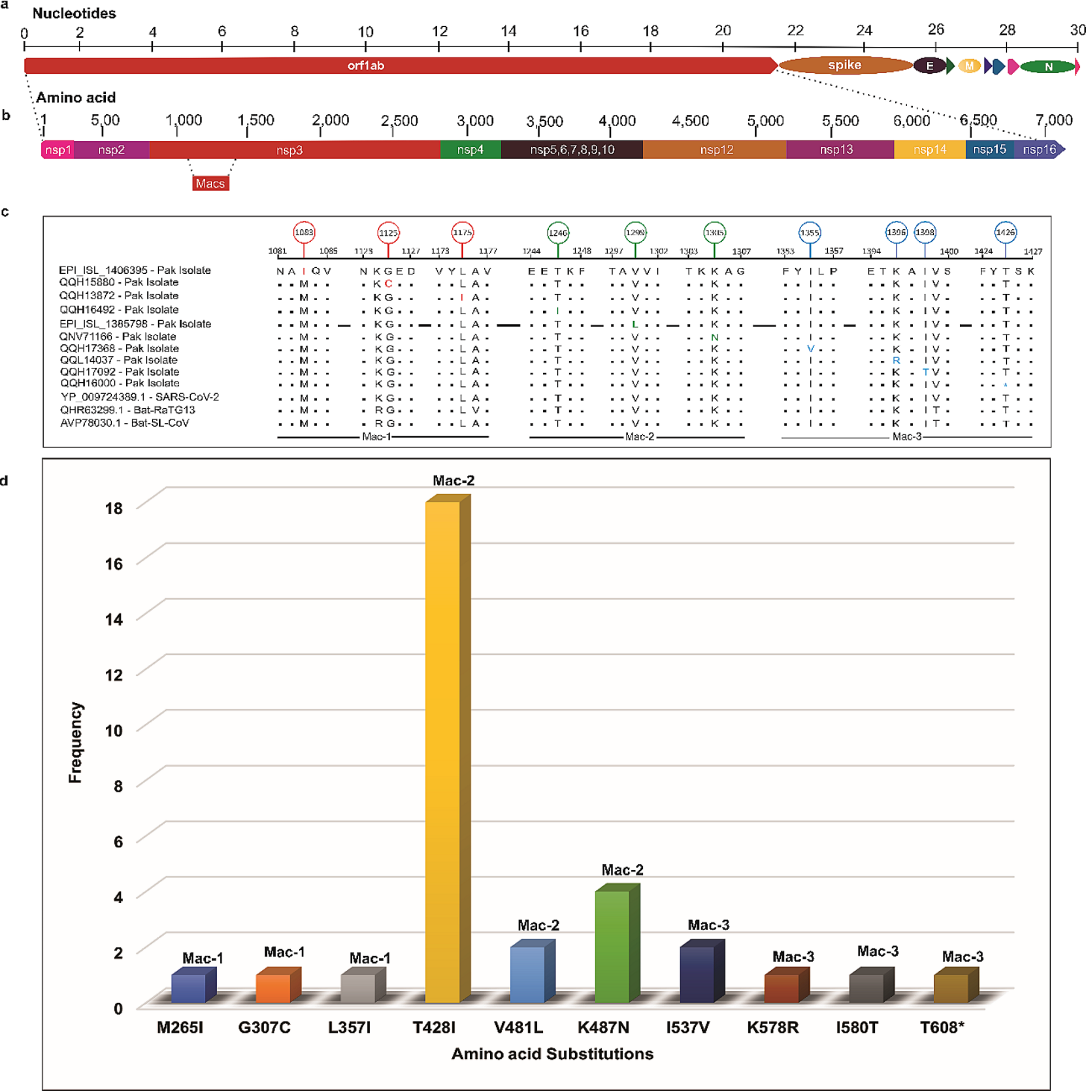
Identification of macrodomain-specific substitutions in polyprotein pp1ab of SARS-CoV-2. **(a)**. Schematic representation of genome of SARS-CoV-2 with numbering above the block refers to the nucleotide positions. Structural proteins including spike (S), envelope (E), membrane (M), and nucleocapsid (N) proteins, as well as the nonstructural proteins (nsps) translated from ORF1ab and the accessory proteins, are represented. **(b).** Schematic representation of 7096-amino acid long polyprotein pp1ab with its 15 sub-proteins (nsp1– nsp10) and (nsp12– nsp16). The positions of macrodomains (Macs: Mac-1, Mac-2, and Mac-3) within Nsp3 are highlighted as a red box demarcated by dotted lines. Numbering above the schematic representation in panel b refers to amino acid positions.**(c).** Comparative Sequence analysis of pp1ab polyprotein of SARS-CoV-2 strains isolated from Pakistan with reference Wuhan strain of SARS-CoV-2 (YP_009725299.1), and closely related Bat-CoVs, i.e., Bat-RaTG13 (QHR63299.1), and Bat-SL-CoV (AVP78030.1). Red, green, and blue colors in the alignment represent the Mac-1, Mac-2, and Mac-3 substitutions, respectively. Panel c, position 1426, the blue asterisk represents a deletion of amino acid.**(d).** The graph shows the frequency of macrodomain-specific amino acid substitutions in the dataset of 203 samples from Pakistan from first wave of pandemic (March 01, 2020, to June 30, 2020). Y-axis represents the frequency of substitutions in Pakistani isolates, whereas the X-axis shows the amino acid substitution position

For comparative sequence analysis, the pp1ab polyprotein sequences of 203 isolates from Pakistan, the reference Wuhan strain of SARS-CoV-2 (YP_009724389.1), and closely related CoVs, namely Bat-SL-CoV (AVP78030.1) and Bat-RaTG13 (QHR63299.1), were aligned using Clustal Omega (Additional File 1: Table S1) [27]. The polyprotein pp1ab sequences of the Wuhan SARS-CoV-2 strain (YP_009724389), Bat-SL-CoV (AVP78030.1), and Bat-RaTG13 (QHR63299.1) were utilized as reference sequences. Manual curation of the alignment facilitated the identification of amino acid substitutions unique to SARS-CoV-2 strains isolated from Pakistan (Additional File 1: Table S3). Moreover, by leveraging GISAID’s CoV server, which is based on viral sequences from the GISAID EpiCoV database, the prevalence of these amino acid substitutions were determined by using previously reported SARS-CoV-2 data (Fig. 2) [21].

To predict the thermodynamic state function ΔΔG (Gibbs free energy) associated with amino acid substitutions from the corresponding reference protein to its mutant version, we used Site Directed Mutator (SDM) server that employs a set of environment-specific substitution tables constrained by conformational information (Additional File 1: Table S5) [28]. Furthermore, the potential physicochemical impact of each substitution on protein structure and function was assessed using the BLOSUM-62 substitution matrix (Additional File 1: Table S5) [29].

### Structural analysis

**Fig. 3:**
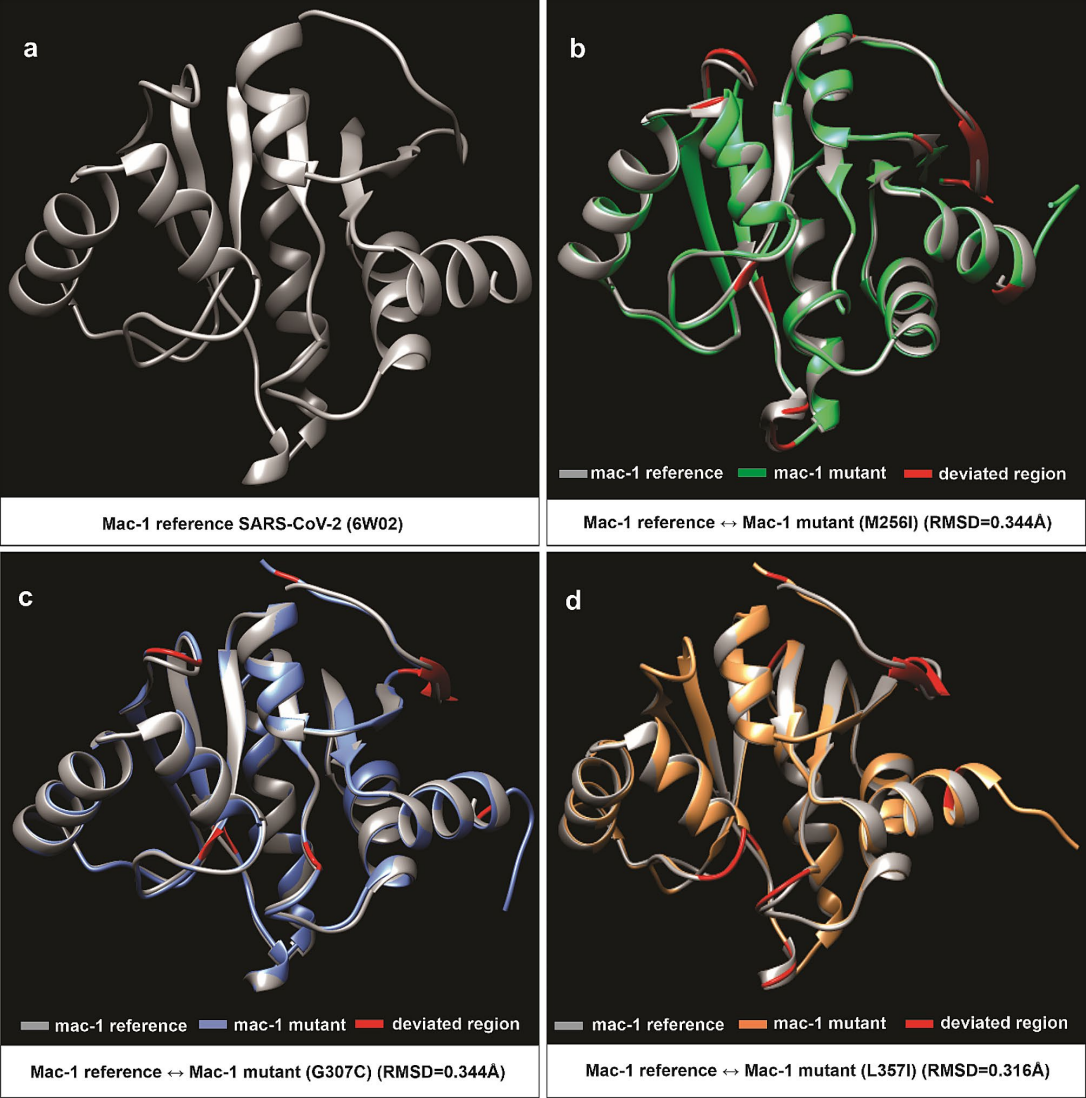
Protein structural analysis of Mac-1 specific amino acid substitutions within Nsp3 of SARS-CoV-2 from Pakistani Isolates. Comparison of 3-dimensional conformations of SARS-CoV-2 macrodomain-1 with their corresponding mutant versions. **(a)** Wild-type structure of Mac-1 (PDB entry: 6W02). **(b-d)** Superimposed structure of wild-type (PDB entry: 6W02) and mutant versions of Mac-1 (M265I: EPI_ISL_1406395, G307C: QQH15880, and L357I: QQL13872). Descriptions of color codes are given in the panel. Deviated residues in terms of backbone torsion angles (Φ°, Ψ°) are shown in red color. Structural deviations are examined by RMSD values and given in the panel **b**, **c**, and **d**. Note. Primary sequence and secondary structural level details for comparisons in **a–d** are given in Additional File 1: Tables S5

**Fig. 4:**
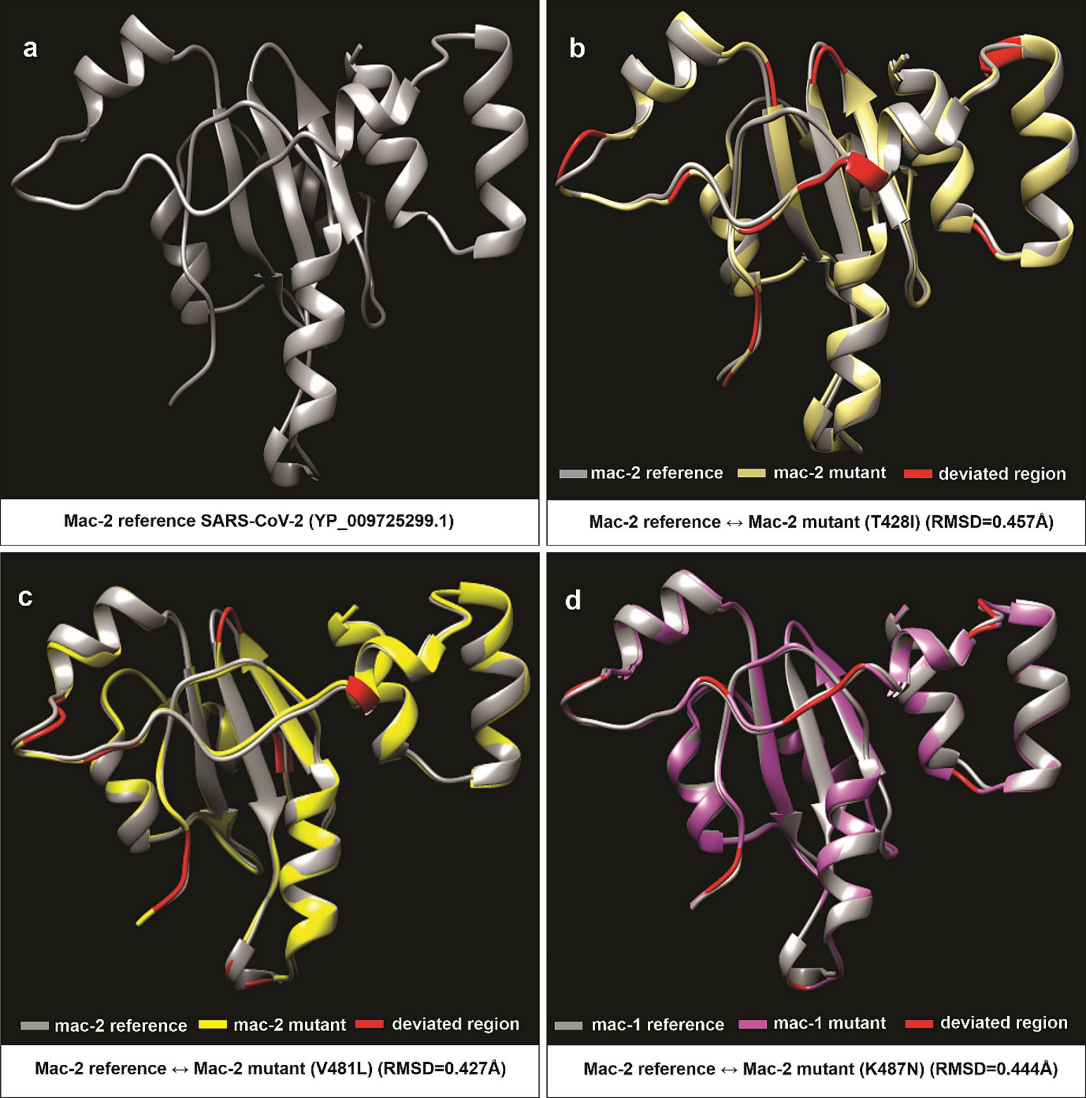
Protein structural analysis of Mac-2 specific amino acid substitutions within Nsp3 of SARS-CoV-2 from Pakistani Isolates. Comparison of 3-dimensional conformations of SARS-CoV-2 macrodomain-2 with their corresponding mutant versions. **(a)** Predicted structure of wild-type Mac-2 (YP_009725299.1). **(b-d)** Superimposed structure of wild-type Mac-2 (YP_009725299.1), and mutant versions of Mac-2 (T428I: QQH16492, V481L: EPI_ISL_1385798, and K487N: QNV71166). Descriptions of color codes are given in the panel. Deviated residues in terms of backbone torsion angles (Φ°, Ψ°) are shown in red color. Structural deviations are examined by RMSD values and given in the panel **b, c**, and **d**. Note. Primary sequence and secondary structural level details for comparisons in **a–d** is given in Additional File 1: Tables S5

To predict protein 3D structures, a comparative modeling approach was employed using the Modeller and Robetta programs [30–32]. Specifically, the crystallographic structure of SARS-CoV-2 Macrodomain-1 (Mac-1) (PDB ID: 6W02) was acquired from the RCSB databank [33]. Additionally, Robetta was used to predict the reference structure of Macrodomain-2 (Mac-2) [32]. Subsequently, corresponding reference structures were utilized for predicting the protein structures of the mutant versions of Mac-1 (M265I, G307C, L357I) and Mac-2 (T428I, V481L, K487N) through Modeller (Figs. 3 and 4) [31]. The protein structures were scrutinized via DOPE score (Discrete Optimized Protein Energy score). Furthermore, the YASARA energy minimization server was employed to perform energy minimization, enhancing the overall quality of the predicted structures [34]. To validate the predicted structures, ERRAT and Rampage Ramachandran plot analysis were conducted [35, 36]. To evaluate the structural alignment between reference and mutant protein structures, the Chimera software was employed, and determined the root mean square deviation (RMSD) values (Figs. 3 and 4) [37, 38]. In summary, a combination of predictive tools, energy minimization, and quality assessment measures were utilized to derive accurate 3D protein structures for further analysis. This approach facilitated the examination of potential structural variations caused by the identified mutations.

### Interaction analysis

**Fig. 5:**
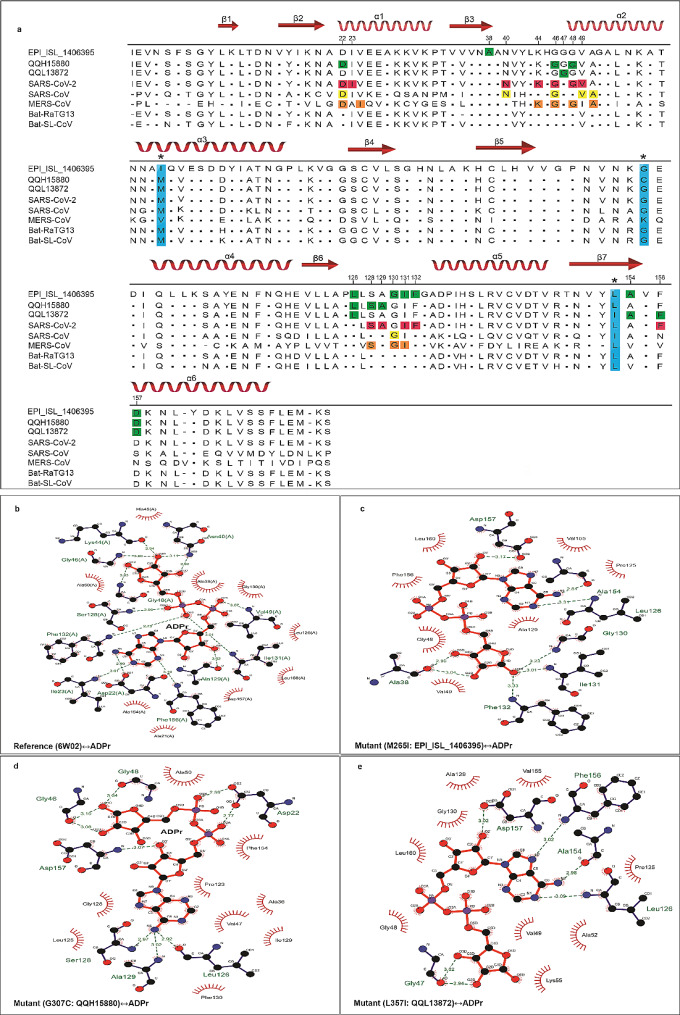
Sequence comparison of Mac-1 and its capacity to bind ADPr. **(a)** Comparison of mutant Mac-1 sequence from Pakistani SARS-CoV-2 isolates (EPI_ISL_1406395, QQH15880, and QQL13872) to that of Mac-1 sequence from reference Wuhan strain of SARS-CoV-2 (PDB entry: 6W02), SARS-CoV (PDB entry: 2FAV), MERS-CoV (PDB entry: 5HOL), Bat-SL-CoV (AVP78030.1), and Bat-RatG13 (QHR63299.1). Secondary structure elements are shown in red at the top of the alignment. In alignment, amino acid substitutions within Mac-1 of Pakistani SARS-CoV-2 isolate is highlighted in blue with an asterisk (*) symbol. The amino acid residues of reference SARS-CoV-2 (PDB entry: 6W02) and its Mac-1 Mutant versions (M265I, G307C and L357I) that form H-bond with the ADP-ribose (ADPr) are highlighted as pink and green, respectively. ADPr interacting residues of SARS-CoV(PDB entry: 2FAV) and MERS-CoV (PDB entry: 5HOL) are highlighted in yellow and orange, respectively and were adopted from previously published work [15].**(b-e)** The LigPlot diagram depicting the 2D interaction pattern of wild-type (PDB entry: 6W02) and Mutant (M265I, G307C and L357I) Mac-1 with ADPr. Legend: thick red lines, ADP ribose ligand; thin black lines, Mac-1 amino acid residue; semi circles with radiating lines represent residues involved in hydrophobic interactions. The Mac-1 residues that form an H-bond with ADPr are represented as green dashed lines in panel **b-e**

To perform a comparative binding analysis between the wild-type and mutant versions of Mac-1 and Adenosine diphosphate ribose (ADPr), the crystallographic structure of SARS-CoV-2 Mac-1 (6W02) was accessed from the RCSB databank [32]. For the docking purpose, AutoDock Vina was utilized [39]. The docking scores facilitated the identification of the most favorable docking compounds. Subsequently, the interactions occurring between critical residues of Mac-1 and ADP-ribose protein was visualized by using Ligplot (Fig. 5b-e) [40]. This analysis provided insights into the molecular interactions that contribute to the binding of Mac-1 with ADPr.

### Molecular dynamic simulation of the wild-type and mutant Mac1-ADPr complex

To explore the dynamic binding features for wild-type and mutant Mac-1 (M265I, G307C, L357I) with ADP-Ribose all-atoms biomolecular simulation was performed using FF14SB force-field in AMBER20 [41]. The system was solvated using TIP3P water box and neutralized the system by adding counter ions [42]. Gentle energy minimization protocol was carried out by employing steepest descent algorithm [43] and the conjugate gradient algorithm at 12,000 and 6000 steps, respectively to eliminate unacceptable steric clashes [44]. Next heating of system at 300 K and weak restraint was used to equilibrate the system at constant pressure of 1 atm and then equilibrated without any restraint. Finally, the 100 ns (ns: nanosecond, that is one billionth of a second) MD production step was performed. Particle Mesh Ewald (PME) algorithm was used to compute long-range interactions [45]. Covalent bond parameterization was performed with the SHAKE algorithm [46]. GPU accelerated simulation using PMEMD. CUDA was used for all the processes. Post-MD trajectories were subjected to thermodynamic stability evaluation, residual flexibility, and structural compactness analysis using CPPTRAJ package of AMBER20 [47].

### Binding free energy calculations

In order to estimate the real binding free energy of Wild-type and mutant Mac-1 (M265I, G307C, L357I)-ADPr complexes, we used MMPBSA.PY script [48]. A 100 ns trajectory having 500 structures were used to calculate the Binding free energy (BFE) using the following equation:

$$ \varDelta {G}_{bind}=\varDelta {G}_{complex}-\left[\varDelta {G}_{receptor}+\varDelta {G}_{ligand}\right]$$

Each term in the binding free energy was estimated using the following equation:

$$ G={G}_{bond}+{G}_{ele}+{G}_{vdW}+{G}_{pol}+{G}_{npol}$$

In the above equation G_bond_, G_ele_, and G_vdW_ represent the bonded, electrostatic, and Vander Waals interactions, respectively. Whereas G_pol_ and G_npol_ are polar and nonpolar solvated free energies, which are calculated by the generalized born (GB) implicit solvent method with the solvent-accessible surface area SASA term.

## Results

### Phylogenetic and comparative sequence analysis of pp1ab of SARS-CoV-2 isolates from Pakistan

This study delved into the genetic variability of polyprotein pp1ab, also known as ORF1ab, sequences within the Pakistani population during the initial wave of the pandemic. To achieve this, all available pp1ab sequences from SARS-CoV-2 strains sampled in Pakistan between March 01, 2020, and June 30, 2020, encompassing the first pandemic wave, were compiled. A total of 203 pp1ab polyprotein sequences, each consisting of 7096 amino acids, were gathered from sources such as NCBI, GISAID, and the 2019 Novel Coronavirus Resource (2019-nCoVR) (Additional File 1: Table S1) [20–23]. By conducting phylogenetic analysis, the evolutionary relationships between SARS-CoV-2 strains circulating within Pakistan and other bat CoVs, including α-CoV, β-CoV, γ-CoV, and δ-CoV were assessed (Fig. 1, Additional File 2: Fig. S1). Subsequently, we scrutinized the variations in these pp1ab polyprotein sequences concerning the reference sequences of the Wuhan strain (YP_009724389.1) and closely related bat-CoVs like bat-RaTG13 (QHR63299.1) and bat-SL-CoV (AVP78030.1) (Fig. 2 a, b; Additional File 1: Tables S1) [2]. This comparative sequence analysis unveiled a total of 179 amino acid differences in pp1ab among SARS-CoV-2 isolates from Pakistan (Additional File 1: Table S3). A majority of these substitutions (38 out of 179) were located within the Nsp3 protein of pp1ab (Additional File 1: Table S3). Nsp3 encodes three consecutive macrodomains: macrodomain-1 (Mac-1), macrodomain-2 (Mac-2), and macrodomain-3 (Mac-3) (Fig. 2 b) [49]. Among the 38 amino acid substitutions detected within Nsp3, at least 10 were localized within the macrodomains (Fig. 2 c, d; Additional File 1: Table S3, S4). Of these macrodomain-specific substitutions, Mac-1 (M265I, G307C, L357I) and Mac-2 (T428I, V481L, K487N) each harbored three substitutions, while Mac-3 contained four amino acid substitutions (I537V, K578R, I580T, T608*) (Fig. 2 c, d; Additional File 1: Tables S4, S5). In our dataset, which encompasses all SARS-CoV-2 genomes collected during the initial wave of the pandemic in Pakistan (from March 01, 2020, to June 30, 2020), specific amino acid substitutions within Mac-1 were detected in one sequence each for M265I, G307C, and L357I (Additional File 1: Table S1 and Table S4). Similarly, variations T428I, V481L, and K487N within Mac-2 were identified in 22, 2, and 4 sequences, respectively (Additional File 1: Table S4). Additionally, substitutions unique to Mac-3, namely I537V, K578R, I580T, and T608*, were found in 2, 1, 1, and 1 sequence, respectively (Additional File 1: Table S4).

Subsequently, GISAID data was analyzed to ascertain the presence of these ten mutations (Pakistan-specific Macrodomains mutations) among SARS-CoV-2 genomes sequenced during subsequent pandemic waves in Pakistan (from July 1, 2020, to February 2024) (https://www.gisaid.org/). Notably, the amino acid substitutions M265I, G307C, and L357I within Mac-1 were detected in 0, 1, and 1 sequence, respectively (https://www.gisaid.org/). Similarly, T428I, V481L, and K487N within Mac-2 were observed in 13, 2, and 4 sequences, respectively. The Mac-3 substitutions I537V, K578R, I580T, and T608* were found in 0, 3, 0, and 0 sequences, respectively (https://www.gisaid.org/).

Further examination of GISAID data up to February 2024 aimed to assess the global prevalence of these macrodomain-specific substitutions in the SARS-CoV-2 genome [21]. This global analysis revealed the amino acid substitutions M265I, G307C, and L357I within Mac-1 in 5,270, 23,375 and 312 sequences, respectively. The substitutions T428I, V481L, and K487N within Mac-2 were found in 32,236, 3,892 and 3,826 sequences, respectively. Moreover, the Mac-3 specific substitutions I537V, K578R, I580T, and T608* were identified in 412, 565, 218 and 649 sequences, respectively (https://www.gisaid.org/).

Moving forward, the functional implications of the identified amino acid substitutions within the macrodomains of SARS-CoV-2 strains were accessed by predicting the thermodynamic-state function [28]. To achieve this, we calculated the Gibbs free energy (ΔΔG) for substitutions concerning the reference sequence of the Wuhan strain (ΔΔG CoV2_reference (YP_009724389.1)_ → CoV2_mutant_) [28]. Four substitutions, namely L357I in Mac-1, T428I in Mac-2, and I537V and K578R in Mac-3, exhibited stabilizing effects (ΔΔG > 0.0), while all other substitutions were found to destabilize the protein structure (ΔΔG < 0.0) (Additional File 1: Table S5). Additionally, analyses of physicochemical properties demonstrated that all amino acid substitutions within macrodomains of SARS-CoV-2 strains isolated from Pakistan, except K487N in Mac-2, were of the radical type, implying their biological significance (Additional File 1: Table S5) [29].

### Comparative structural analysis of macrodomains

Given the established role of macrodomains in countering the host’s antiviral response, our focus turned to evaluating the structural impact of amino acid substitutions through modeling mutant versions of the macrodomains (Mac-1 and Mac-2). To achieve this, the crystal structure of the reference sequence of Mac-1 (PDB id: 6W02) was acquired from the RCSB PDB. Furthermore, we modeled the reference sequence of Mac-2 (YP_009725299.1), as well as Mac-1 mutants (M265I: EPI_ISL_1406395, G307C: QQH15880, and L357I: QQL13872), and Mac-2 mutants (T428I: QQH16492, V481L: EPI_ISL_1385798, and K487N: QNV71166) using a comparative modeling approach [30–33]. The superimposition of the wild-type (reference sequence; PDB id: 6W02) Mac-1 protein structure on its mutant versions (M265I: EPI_ISL_1406395, G307C: QQH15880, and L357I: QQL13872), and the superimposition of the wild-type Mac-2 protein structure (derived from the reference sequence YP_009725299.1) on its mutant versions (T428I: QQH16492, V481L: EPI_ISL_1385798, K487N: QNV71166) were carried out, and the structural deviations were assessed using RMSD values (Figs. 3a-d and 4a-d; Additional File 1: Table S6) [38]. The calculated RMSD values between wild-type Mac-1 (PDB ID: 6W02) and its mutants M265I (EPI_ISL_1406395), G307C (QQH15880), and L357I (QQL13872) were 0.344Å, 0.344Å, and 0.316Å, respectively (Fig. 3 b-d). Correspondingly, the RMSD values of the superimposed structures of wild-type Mac-2 (YP_009725299.1) and its mutant structures T428I (QQH16492), V481L (EPI_ISL_1385798), and K487N (QNV71166) were 0.457Å, 0.427Å, and 0.444Å, respectively (Fig. 4b-d).

In an effort to delve deeper into the specific differences among the superimposed structures, we conducted an analysis of secondary structural elements (SSEs). Our 3D superimposition of wild-type and mutant macrodomains (both Mac-1 and Mac-2) unveiled significant shifts in backbone torsion angles and secondary structure elements (SSEs) resulting from amino acid substitutions at the primary sequence level (Figs. 3b-d and 4b-d). We observed transitions from loops to secondary structure elements, such as helices and beta-sheets (Additional File 1: Table S6). For instance, the wild-type Mac-1 (PDB id: 6W02) of SARS-CoV-2 exhibited approximately 65% of its residues within SSEs, while its mutant versions (M265I: EPI_ISL_1406395, G307C: QQH15880, and L357I: QQL13872) contained about 56%, 59%, and 59% of residues in SSEs, respectively (Additional File 1: Table S6). Similarly, the wild-type Mac-2 (YP_009725299.1) contained around 51% of residues in SSEs, whereas its mutant versions (T428I: QQH16492, V481L: EPI_ISL_1385798, and K487N: QNV71166) comprised approximately 52%, 50%, and 49% of residues in SSEs, respectively (Additional File 1: Table S6). Thus, amino acid substitutions within the macrodomains of SARS-CoV-2 induced significant structural shifts within both Mac-1 and Mac-2 in three-dimensional space (Figs. 3b-d and 4b-d; Additional File 1: Table S6).

### Molecular docking analysis of Macrodomain-1 of SARS-CoV-2 with ADP-ribose

Mac-1, a key player in hydrolyzing and binding Adenosine diphosphate ribose (ADPr) from the host target protein, is a significant biochemical feature of SARS-CoV-2’s strategy to counter the host’s antiviral response involving ADP-ribosylation [50]. Thus, the relationship between protein conformational changes induced by amino acid substitutions (M265I, G307C, L357I) and the potential functional alterations in Mac-1 was evaluated. To this end, molecular docking was performed to assess the binding affinity of ADPr to Mac-1. Our structural analyses revealed noticeable disparities in ADPr binding patterns between the wild-type and mutant versions of Mac-1 (M265I, G307C, L357I) (Fig. 5b-e). The reference Mac-1 structure (PDB id: 6W02) exhibited a more favorable ADPr binding efficiency (-9.9 kcal/mol) compared to its mutant counterparts, M265I (-7.3 kcal/mol), G307C (-6.6 kcal/mol), and L357I (-6.7 kcal/mol) (Additional File 1: Table S7).

To decipher the molecular foundation of these differences in binding energies, we further scrutinized the ADPr binding clefts of both the wild-type Mac-1 (6W02) and its mutants (M265I, G307C, L357I) (Fig. 5b-e; Additional File 1: Table S7). The wild-type Mac-1 (6W02) established 12 hydrogen bonds with ADPr (involving amino acid residues Asp22, Ile23, Asn40, Phe156, Ala129, Ile131, Phe132, Val49, Ser128, Gly48, Gly46, and Lys44). In contrast, Mac-1 mutant version M265I formed 7 hydrogen bonds (Ala38, Leu126, Gly130, Ile131, Phe132, Ala154, Asp157), 7 hydrogen bonds by Mac-1 mutant version G307C (Asp22, Leu126, Ala129, Ser128, Asp157, Gly47, and Gly48) and 5 hydrogen bonds by Mac-1 mutant version L357I (Gly46, Leu126, Ala154, Asp157, Phe156) with ADPr (Fig. 5b-e; Additional File 1: Table S7). Notably, Asp-22, a conserved residue among macrodomains of coronaviruses (CoVs), played a pivotal role in ADPr binding by forming a hydrogen bond with the N6-atom of the pyrimidine ring in the adenine moiety [15]. The comparative examination of the ADPr binding cleft illustrated that while the reference Mac-1 structure (PDB id: 6W02) engaged in this hydrogen bond, Mac-1 mutants M265I and L357I lacked such an interaction. Additionally, Mac-1 mutant G307C’s interaction with ADPr involved the diphosphate region rather than the adenine moiety (Fig. 5a-e). Collectively, it seems that the conformational epistatic effects of M265I, G307C, and L357I substitutions may have compromised Mac-1’s ability in the Pakistani isolates to effectively counteract the host’s antiviral ADPr activity, in contrast to the reference Mac-1 (PDB id: 6W02) [51].

### Molecular dynamic simulation of the wild-type and mutant Mac1-ADPr complex

**Fig. 6:**
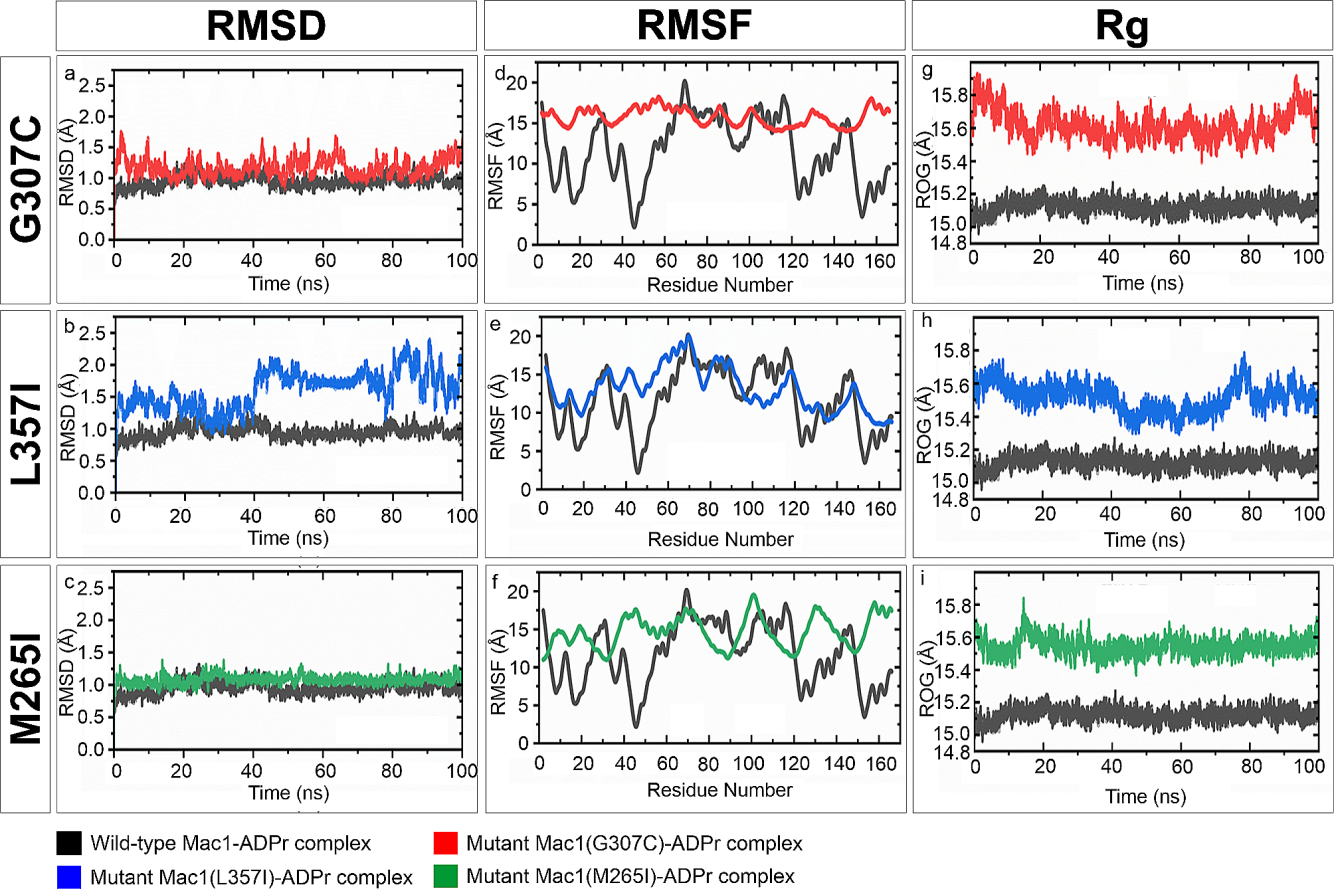
RMSD, RMSF, and Rg plots of wild type and mutant Mac1 (M265I, G307C, L357I)-ADPr complexes obtained through 100 ns MD simulation trajectories. Panels (**a-c**) display the RMSD of the wild-type Mac1-ADPr complex in comparison with each mutant Mac1-ADPr complex. Panels (**d-f**) illustrate the RMSF of the wild-type Mac1-ADPr complex and each mutant Mac1-ADPr complex. Panels (**g-i**) showcase the radius of gyration of the wild-type Mac1 and each mutant Mac1-ADPr complex. Each complex is color-coded: wild-type Mac1-ADPr complex in black, mutant Mac1 (G307C)-ADPr complex in red, mutant Mac1 (L357I)-ADPr complex in blue, and mutant Mac1 (M265I)-ADPr complex in green. RMSD: Root Mean Square Deviation, RMSF: Root Mean Square Fluctuation, Rg: Radius of gyration

In order to gain deeper insights into the dynamic behaviors and binding variations induced by mutant versions of Mac-1 (M265I, G307C, L357I), molecular dynamics (MD) simulations were performed (Fig. 6a-i). This advanced approach enabled us to delve into the structural stability, flexibility, and compactness of both the wild-type and mutant Mac1 (M265I, G307C, L357I)-ADPr complexes. Through simulations time of 100 ns trajectories, the goal was to attain a more comprehensive understanding while ensuring alignment with the methods elucidated in the preceding section (Molecular Docking Analysis of SARS-CoV-2 Macrodomain-1 and ADP-Ribose Interaction) (Fig. 6a-i).

To assess the comparative conformational stability of the wild-type Mac1-ADPr complex and the mutant versions of Mac1 (M265I, G307C, L357I)-ADPr complexes, Root Mean Square deviations (RMSD) from the Cα backbone was calculated (Fig. 6a-c). The resulting RMSD plots, illustrated in Fig. 6, highlighted notable differences between the wild-type Mac1-ADPr complex and the mutant iterations (M265I, G307C, L357I)-ADPr complexes. Notably, the RMSD values within the mutant Mac1 (M265I)-ADPr complex were slightly higher in contrast to the wild-type Mac1-ADPr complex (Fig. 6c). Conversely, the Mac-1 mutants Mac1 (G307C)-ADPr and Mac1 (L357I)-ADPr displayed considerably higher RMSD [suggesting weaker interactions than mutant Mac1(M265I)-ADPr)] in comparison to the wild-type complex (Fig. 6a, b).

Additionally, within the realm of macromolecular interactions, local flexibility and thermal stability were evaluated through root mean-square fluctuations (RMSF) derived from molecular dynamics simulations [52]. Elevated RMSF values generally signify augmented flexibility and correspondingly less stable interactions. Our observations indicated that the mutant versions of Mac1 (M265I, G307C, L357I)-ADPr complexes exhibited relatively higher RMSF when compared to the wild-type Mac1-ADPr complex (Fig. 6d-f). This trend strongly suggests the potential for unstable binding interactions within the Mac1 mutant complexes (M265I, G307C, L357I) (Fig. 6d-f).

Furthermore, further insight into structural characteristics was gained by calculating the Radius of Gyration (Rg) as a measure of protein structure compactness throughout the simulation [53]. Distinctive Rg patterns emerged between the wild-type Mac1-ADPr complex and the mutant versions of Mac1 (M265I, G307C, L357I)-ADPr complexes (Fig. 6g-i). Notably, while the average Rg value for the wild-type Mac-1 was 15.0 Å, the mutant versions (M265I, G307C, L357I) displayed slightly higher average Rg values of 15.4 Å, 15.6 Å, and 15.4 Å, respectively (Fig. 6g-i). This discrepancy highlights the notably tighter and more stable packing within the wild-type Mac1-ADPr complex compared to the mutant iterations (M265I, G307C, L357I)-ADPr complexes (Fig. 6g-i).

For each complex [(wild-type and mutant Mac1(M265I, G307C, L357I)-ADPr complexes)], the binding free energy was evaluated using 500 structural frames obtained from MD simulations (Additional File 1: Table S8) [48]. A detailed comparison of the energetic profiles of the complexes is presented in Additional File 1: Table S8. Collectively, the total free energy of binding for the wild-type Mac1-ADPr complex is more favorable (-36.1127 kcal/mol) than the binding energies of Mac-1 mutants: Mac1 (M265I)-ADPr complex (-22.3857 kcal/mol), Mac1 (G307C)-ADPr complex (-17.9755 kcal/mol), and Mac1 (L357I)-ADPr complex (-15.5715 kcal/mol) (Additional File 1: Table S8). These energy profiles suggest the tighter binding of the wild-type Mac-1 than the mutant version of Mac-1 (M265I, G307C, L357I) with ADPr.

In summary, the data acquired from the initial rigid molecular docking analysis was consistently reinforced through the application of more advanced molecular dynamics (MD) simulations (Fig. 6a-i, Additional File 1: Table S8). This congruence amplifies our confidence in the reliability and consistency of our findings concerning the impact of the studied amino acid substitutions on Mac-1’s binding interactions with ADPr.

## Discussion

The initial wave of COVID-19 in Pakistan commenced in March 2020 and concluded in July 2020 [54]. This wave peaked in mid-June with an overall positivity rate that fluctuated between18–23% [54, 55]. Notably, the mortality rate experienced during this inaugural wave in Pakistan was notably lower in comparison to several other countries [16, 17]. As an illustration, Pakistan observed a mortality rate of 2%, whereas Iran, along with several European nations including the UK and Italy, recorded relatively higher mortality rates—namely, 4.68%, 3.43%, and 3.53% respectively [16, 17]. This variance in mortality rates may stem from a multitude of factors, encompassing the genetic foundation of the host, strategies for clinical management and treatment, policies regarding lockdowns, coexisting health conditions, and age demographics [56–59]. The elderly population tends to exhibit an elevated risk of mortality due to COVID-19; however, Pakistan is distinguished by its substantial proportion of youth, constituting around 63% of the total population and aged between 15 and 33 [59, 60]. Nevertheless, it is noteworthy that genetic variations within the SARS-CoV-2 genome can also contribute to the disparities in mortality rates. Numerous investigations have detailed the correlation between genetic modifications in the SARS-CoV-2 genome and mortality rates [11, 18, 61–63]. For instance, the D614G mutation in the spike protein has been linked to augmented mortality rates across various European nations [11, 63]. Conversely, the L37F mutation within Nsp6 is associated with decreased mortality rates in Singapore, Malaysia, South Korea, and various other countries [18]. Within the ambit of the current study, the characterization of genetic variations within the 7096-amino-acid-long pp1ab polyprotein of SARS-CoV-2 strains that were isolated from Pakistan during the inaugural wave of the COVID-19 pandemic was undertaken. The focus within pp1ab lay on the macrodomains encoded by Nsp3, with the aim of discerning their structural evolution and establishing potential links with the mortality rate in Pakistan.

The globular domain of Mac-1, as encoded by Nsp3, encompasses a conserved cleft with an affinity for binding to ADP-ribose (ADPr) [50]. This domain exhibits hydrolase activity, enabling it to remove ADP-ribose from host target proteins—a mechanism employed to counteract the host’s antiviral response of ADP-ribosylation [50]. The de-ADP-ribosylation function of Mac-1 has been linked to the virulence of SARS-CoV-2 and its capacity to evade the host’s immune response [50]. Studies have demonstrated that a reduced ability of Mac-1 to eliminate ADP-ribose from host target proteins results in the attenuation of SARS-CoV-2 infection and heightened vulnerability of coronaviruses to the host’s innate immune response [64–66]. In addition to their enzymatic roles, Mac-2 and Mac-3engage with G-quadruplexes situated in the 3’-untranslated regions of mRNAs encoding proteins involved in the host’s antiviral response—factors related to apoptosis or signal transduction. This interaction leads to the disruption of the host cell’s reaction to viral infection [67, 68]. Furthermore, both Mac-2 and Mac-3, together with the papain-like protease (PL2pro) domain encoded by Nsp3, establish interactions with RCHY1, an E3 ubiquitin ligase. This interaction culminates in the degradation of p53, a pivotal component of the host’s antiviral innate immunity. The Mac-2 and Mac-3-driven degradation of p53 results in the delayed activation of immune genes regulated by p53 [69]. Interestingly, while Mac-1 is conserved across all coronaviruses, Mac-2 and Mac-3 are found specifically in highly pathogenic coronaviruses, including SARS-CoV-2 [50].

In this study, we undertook a comprehensive analysis to elucidate the evolutionary and protein structural aspects of the SARS-CoV-2 strains isolated from Pakistan during the initial wave of COVID-19. Our phylogenetic analysis encompassing the four genera of the subfamily Coronavirinae revealed a divergent pattern, where the δ-CoVs and γ-CoVs formed the earliest cluster, succeeded by α-CoVs, β-OC43-CoVs, β-MERS-CoVs, and β-SARS-CoVs, respectively (Fig. 1; Additional File 2: Fig.S1). Specifically, the phylogenetic tree demonstrated a direct grouping of SARS-CoV-2 isolates from Pakistan’s first wave (March to June) with BatCoV-RaTG13. This clade, consisting of SARS-CoV-2 Pakistani isolates and BatCoV-RaTG13, shared an ancestral branch with the cluster of batSL-CoVs (Fig. 1; Additional File 2: Fig.S1). This pattern reinforces prior findings, indicating that SARS-CoV-2 likely originated from ancestral Bat-CoVs (Fig. 1; Additional File 2: Fig.S1) (Hussain et al. 2020). Moving forward, a comparative analysis was conducted, involving 203 protein sequences of the pp1ab polyprotein of SARS-CoV-2 strains isolated from Pakistan between March 01 and June 30, 2020. These sequences were evaluated against the reference Wuhan strain (YP_009724389.1) and closely related bat-CoVs, namely bat-RaTG13 (QHR63299.1) and bat-SL-CoV (AVP78030.1) as outgroups (Additional File 1: Table S1). Our analysis revealed a total of 10 amino acid differences within the macrodomains of SARS-CoV-2 strains from Pakistan. These substitutions included three in Mac-1 (M265I, G307C, L357I), three in Mac-2 (T428I, V481L, K487N), and four in Mac-3 (I537V, K578R, I580T, T608*) (Fig. 2c, d; Additional File 1: Tables S4, S5).

Through a meticulous analysis of GISAID-based data, noteworthy trend has been discerned. Specifically, the ten macrodomain-specific substitutions identified in the SARS-CoV-2 genomes sampled during the initial pandemic wave exhibited a notable rarity in subsequent pandemic waves, which extended from July 01, 2020, to February 2024. To illustrate, the Mac-1 specific amino acid replacement, M265I, was conspicuously absent in a dataset comprising 6,750 complete genome sequences of SARS-CoV-2 from Pakistan, spanning the period from July 01, 2020, to February 2024. In contrast, the presence of the G307C mutation was observed within one of the Omicron variants (GISAID Accession no: EPI_ISL_16905153), while the L357I mutation manifested in one of the Delta variants (GISAID Accession no: EPI_ISL_5779768). Consequently, the macrodomain-specific amino acid replacements identified in our study are unequivocally associated with the first wave of the pandemic, occurring between March 01, 2020, and June 30, 2020.

However, our scrutiny of post-first wave data has unveiled novel macrodomain variations within the alpha, beta, delta, and omicron variants of SARS-CoV-2 that emerged in Pakistan between July 01, 2020, and February 2024 (Additional File 1: Table S10). These post-first wave SARS-CoV-2 genome data provide compelling evidence of ongoing evolutionary changes within the macrodomains of SARS-CoV-2, potentially exerting a substantial impact on viral fitness variability. Consequently, future studies should delve into conducting a comprehensive examination of the structural and biophysical characteristics of these macrodomain variants that have emerged subsequent to the first wave.

To assess the functional implications of these first wave specific amino acid replacements, 3D superimposition of the macrodomains between the reference Wuhan strain (YP_009724389.1) and the specific variants from Pakistani isolates was performed. The results indicated substantial structural deviations in backbone torsion angles and secondary structural elements, suggesting potential functional significance (Figs. 3b-d and 4b-d; Additional File 1: Table S6). To further probe the potential functional impact of the observed substitutions, rigid molecular docking approach was utilized to determine the binding affinity of ADP-ribose (ADPr) to Mac-1. These analyses revealed that the Mac-1 mutant versions i.e., M265I (-7.3 kcal/mol), G307C (-6.6 kcal/mol), and L357I (-6.7 kcal/mol) binds less efficiently to ADPr compared to the reference Mac-1(6W02) (-9.9 kcal/mol) (Additional File 1: Table S7). In-depth investigation of the binding cleft revealed that these substitutions have altered the hydrogen bonding between Mac-1 cleft and ADPr, both in terms of number and pattern (Fig. 5a-e; Additional File 1: Table S7). For instance, binding cleft of wild-type (reference sequence) Mac-1 (6W02) forms 12 hydrogen bonds with ADPr, whereas Mac-1 mutants M265I (EPI_ISL_1406395), G307C (QQH15880), and L357I (QQL13872) formed only 7,7 and 5 hydrogen bonds with ADPr, respectively (Fig. 4a-e; Additional File 1: Table S7). In previous studies on MERS-CoV/SARS-CoV-2, a conserved Asp residue (Asp20 in MERS-CoV and Asp22 in SARS-CoV-2) within the α1-helix of Mac-1 has been identified as crucial for binding specificity [15]. The side chain of this residue forms a direct contact with ADP-ribose (ADPr) through hydrogen bonding involving the N-6 atom of the pyrimidine ring in the adenine moiety. Our findings in this study align seamlessly with this observation (Fig. 4b). Consequently, this interaction leads to a repositioning of the side chain of Asp20/Asp22 into the adenine cavity, thereby reinforcing ADPr binding and effectively diminishing host antiviral ADPr activity [70]. The conservation of this specific residue (Asp20 in MERS-CoV and Asp22 in SARS-CoV-2) and its binding to the N-6 atom of the ADPr pyrimidine ring is a characteristic feature among the macrodomains found in highly contagious CoVs [15, 50]. In this study, our comparative analysis of the binding cleft has revealed that within Mac-1 mutants—specifically M265I and L357I—Asp22 does not establish any hydrogen bond interactions with ADP-ribose (ADPr) (Fig. 4c, e). On the other hand, in the case of the Mac-1 mutant G307C, Asp22 forms an interaction with the diphosphate group of ADPr instead of the N-6 atom in the adenine moiety (Fig. 4d). This altered interaction may have led to a repositioning of the side chains of Asp22 with respect to the cleft that accommodates the adenine moiety (Fig. 4b-e). Prior research studies have indicated that the failure of this conserved Asp residue to directly bind with the N-6 atom of adenine results in a significantly reduced affinity between the Mac-1 of HCoV 229 and ADPr compared to the MERS-CoV homologue [70]. As a result, it is conceivable that the lower binding affinity observed between the Mac-1 mutants (M265I, G307C, and L357I) and ADPr, in contrast to the wild-type Mac-1, may be attributed to a decreased number of hydrogen bonds and a modified hydrogen-bonding pattern (Fig. 4b-e; Additional File 1: Table S7). Moreover, the results obtained from the MD simulations provide additional support for the notion of increased dynamic stability within the wild-type Mac1-ADPr complex when contrasted with the mutant iterations of Mac1 (M265I, G307C, L357I)-ADPr complexes during the 100ns simulation duration (Fig. 6a-i). These findings were further validated by the calculations of binding free energy derived from the MD simulations. Specifically, these calculations demonstrated a more favorable total free energy for the wild-type Mac1-ADPr complex compared to the mutant forms of Mac1 (M265I, G307C, L357I)-ADPr complexes (Additional File 1: Table S8).

Previously, it has been demonstrated that a diminished capacity or affinity of Mac-1 to eliminate ADPr in CoVs leads to a reduction in virulence and heightened sensitivity to host innate immune responses [15, 70]. Hence, it is reasonable to speculate that the weakened binding of mutant versions of Mac-1 (M265I, G307C, and L357I) to ADPr might have compromised the ability of SARS-CoV-2 isolates from Pakistan (EPI_ISL_1406395, QQH15880, QQL13872) to evade the antiviral ADPr activity within the host, resulting in a decrease in virulence and pathogenicity. Aligning with this hypothesis, it is conceivable to propose that conformational changes in proteins due to subtle genetic variations (such as M265I, G307C, and L357I within the Mac-1 domain of Nsp3) could have manifested as significant disparities in the virulence and pathogenicity of SARS-CoV-2 strains present in different geographical regions. This could account for the lower mortality observed in Pakistan during the initial wave of the COVID-19 pandemic compared to neighboring countries.

The transmission of SARS-CoV-2 among individuals of varying ages, genetic backgrounds, and medical susceptibilities has led to the rapid accumulation of mutations in its genome across different geographical regions. This phenomenon has given rise to multiple viral variants, endowing SARS-CoV-2 with evolved characteristics such as heightened transmissibility, increased disease severity, and enhanced evasion of host immunity [8, 71]. Among these variants, particular attention has been directed toward mutations in the 1273-amino acid Spike glycoprotein (S glycoprotein), given its significant association with changes in SARS-CoV-2’s properties [10]. The Receptor Binding Domain (RBD) within the S1 subunit of the S glycoprotein plays a pivotal role in the initial stages of infection, as it facilitates the entry of the virus into host cells by binding to the membrane-bound angiotensin-converting enzyme 2 (ACE2). This interaction underscores the importance of the RBD in SARS-CoV-2’s infectivity and transmissibility. Numerous mutations in the S protein can enhance the virus’s ability to infect, including immune evasion, heightened expression, and resistance to neutralizing monoclonal antibodies (mAbs) and convalescent serum [72, 73]. As a result, the combination of enhanced transmissibility and immune evasion represents a powerful pathway for a virus to achieve increased fitness and viability. This evolution underscores the dynamic interplay between viral mutations and host responses in shaping the trajectory of the COVID-19 pandemic.

In the dynamic landscape of SARS-CoV-2 evolution, significant attention has centered on the variations within the Spike (S protein) glycoprotein [10, 12, 13]. Nevertheless, it remains vital to acknowledge the pivotal role of non-S mutations in sculpting the repertoire of SARS-CoV-2 variants, orchestrating heightened transmission across nations and continents. Counterintuitively, some non-S mutations may exert a detrimental influence on viral fitness, as evidenced by previously published research [14, 15, 73]. For instance, non-S mutations like R203K/G204R within the nucleocapsid protein can bolster transmission and virulence while enhancing SARS-CoV-2’s overall fitness [74]. Additionally, non-S mutations, encompassing the non-structural proteins (NSPs) encoded by the pp1ab polyprotein, influence the virulence dynamics of SARS-CoV-2. Notably, the T429I substitution in non-structural protein-4 (NSP4) enhances replication capacity, improves evasion mechanisms against host immune responses, and augments the infectivity of SARS-CoV-2 [75]. Similarly, the triple deletion ΔSGF in non-structural protein-6 (NSP6) significantly impacts host-pathogen interactions, resulting in discernible alterations in the virulence profile of SARS-CoV-2 [76].

In our study, focus was placed on characterizing a non-S mutation within the conserved macrodomain-1 of SARS-CoV-2, sampled from Pakistan during the initial wave of the COVID-19 pandemic. Our findings reveal that specific amino acid replacements (M265I, G307C, and L357I) within the Mac-1 region exert detrimental effects on SARS-CoV-2’s fitness through conformational epistatic interactions (Fig. 2). These observations align with previous studies, such as the identification of a 29-nucleotide deletion in the ORF8 of SARS-CoV, which exhibited a negative impact on viral fitness and reduced epidemic risks [77]. Drawing from these examples, it’s plausible to speculate that naturally occurring mutations within functionally critical segments of SARS-CoV-2 could harbor significant yet overlooked detrimental consequences for viral fitness. These effects may contribute to a reduction in estimated virulence and mitigate the health burden associated with the COVID-19 outbreak.

## Conclusion

While the COVID-19 pandemic has been significantly controlled through widespread vaccination efforts globally, and the impact of SARS-CoV-2 on human health is on a downward trajectory, the ongoing investigation and monitoring of SARS-CoV-2’s molecular and genetic pathogenic mechanisms remain crucial. Such endeavors not only aid in addressing current challenges but also hold the key to better managing potential viral-based pandemic events in the future. In this regard, comprehensive biological characterizations of these variants offer invaluable insights into the transmission dynamics and infectivity of SARS-CoV-2, paving the way for informed strategies encompassing vaccines, antiviral agents, and precision medicine, particularly tailored to populations harboring genetic risk variants.

### Electronic supplementary material

Below is the link to the electronic supplementary material.

Supplementary Material 1

Supplementary Material 2

## Data Availability

Not applicable.

## Abbreviations

COVID-19: Coronavirus Disease 2019
SARS-CoV-2: Severe acute respiratory syndrome coronavirus 2
Nsp3: Non-structural protein 3
ADPr: Adenosine diphosphate ribose
WHO: World Health Organization
ORF: Open Reading Frame
NCBI: National Center for Biotechnology Information
GISAID: Global Initiative on Sharing All Influenza Data
2019-nCoVR: 2019 Novel Coronavirus Resource
DOPE score: Discrete Optimized Protein Energy score
RMSD: Root mean square deviation
Mac-1: Macrodomain-1
Mac-2: Macrodomain-2
Mac-3: Macrodomain-3
SSEs: Secondary Structural Elements
PL2pro: Papain-like protease
MERS-CoV: Middle East respiratory syndrome coronavirus
CoVs: Coronaviruses
RBD: Receptor Binding Domain
ACE2: Angiotensin-converting enzyme 2
RMSF: Root mean square fluctuation
Rg: Radius of gyration
