# Supplementary material for: Unraveling the genetic variations underlying virulence disparities among SARS-CoV-2 strains across global regions: insights from Pakistan
Source: Virol J. 2024 Mar 6;21:55. doi: 10.1186/s12985-024-02328-8 (PMC10916261; doi:10.1186/s12985-024-02328-8)
Supplement: Supplementary file 1 — Supplementary Material 1 [file 12985_2024_2328_MOESM1_ESM.pdf]

Supplementary data for:

Unraveling the Genetic Variations Underlying Virulence Disparities Among SARS-CoV-2 Strains: Insights from Pakistan

Momina Jabeen[a], Shifa Shoukat[a], Huma Shireen[a], Yiming Bao[b, c], Abbas Khan[d,e], & Amir Ali Abbasi[a]*

[a]National Center for Bioinformatics, Program of Comparative and Evolutionary Genomics, Faculty of Biological Sciences, Quaid-i-Azam University, Islamabad 45320, Pakistan

[b]National Genomics Data Center & CAS Key Laboratory of Genome Sciences and Information, Beijing Institute of Genomics, Chinese Academy of Sciences, and China National Center for Bioinformation, Beijing 100101, China

[c]University of Chinese Academy of Sciences, Beijing 100101, China

[d]Department of Bioinformatics and Biological Statistics, School of Life Sciences and Biotechnology, Shanghai Jiao Tong University, Shanghai 200240, China.

[e]School of Medical and Life Sciences, Sunway University, Sunway City, Malaysia.

*Corresponding author (A.A.A) Email: abbasiam@qau.edu.pk

Email Addresses

MJ: E-mail: mominajabeen999@gmail.com

SS: E-mail:shifabinteshoukat@gmail.com

HS: E-mail: humashireen.qau@gmail.com

YB: E-mail: baoym@big.ac.cn

AK: E-mail: abbaskhan@sjtu.edu.cn

AAA*: E-mail:abbasiam@qau.edu.pk

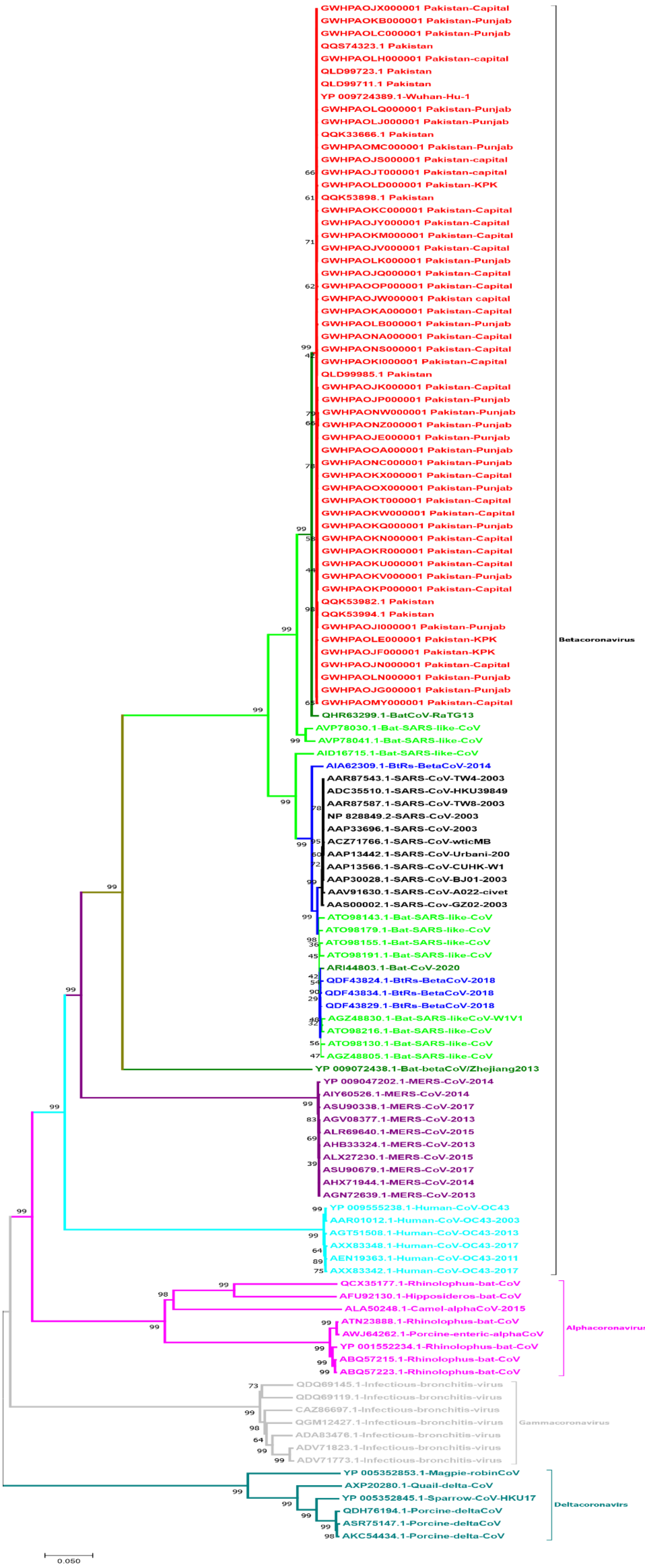
GWHPAOJX000001 Pakistan-Capital
GWHPAOKB000001 Pakistan-Punjab
GWHPAOLC000001 Pakistan-Punjab
QQS74323.1 Pakistan
GWHPAOLH000001 Pakistan-capital
QLD99723.1 Pakistan
QLD99711.1 Pakistan
YP 009724389.1-Wuhan-Hu-1
GWHPAOLQ000001 Pakistan-Punjab
GWHPAOLJ000001 Pakistan-Punjab
QQK33666.1 Pakistan
GWHPAOMC000001 Pakistan-Punjab
GWHPAOJS000001 Pakistan-capital
GWHPAOJT000001 Pakistan-capital
GWHPAOLD000001 Pakistan-KPK
QQK53898.1 Pakistan
GWHPAOKC000001 Pakistan-Capital
GWHPAOJY000001 Pakistan-Capital
GWHPAOKM000001 Pakistan-Capital
GWHPAOJV000001 Pakistan-Capital
GWHPAOLK000001 Pakistan-Punjab
GWHPAOJQ000001 Pakistan-Capital
GWHPAOOP000001 Pakistan-Capital
GWHPAOJW000001 Pakistan capital
GWHPAOKA000001 Pakistan-Capital
GWHPAOLB000001 Pakistan-Punjab
GWHPAONA000001 Pakistan-Capital
GWHPAONS000001 Pakistan-Capital
GWHPAOKI000001 Pakistan-Capital
QLD99985.1 Pakistan
GWHPAOJK000001 Pakistan-Capital
GWHPAOJP000001 Pakistan-Punjab
GWHPAONW000001 Pakistan-Punjab
GWHPAONZ000001 Pakistan-Punjab
GWHPAOJE000001 Pakistan-Punjab
GWHPAOOA000001 Pakistan-Punjab
GWHPAONC000001 Pakistan-Punjab
GWHPAOKX000001 Pakistan-Capital
GWHPAOOX000001 Pakistan-Punjab
GWHPAOKT000001 Pakistan-Capital
GWHPAOKW000001 Pakistan-Capital
GWHPAOKQ000001 Pakistan-Punjab
GWHPAOKN000001 Pakistan-Capital
GWHPAOKR000001 Pakistan-Capital
GWHPAOKU000001 Pakistan-Capital
GWHPAOKV000001 Pakistan-Punjab
GWHPAOKP000001 Pakistan-Capital
QQK53982.1 Pakistan
QQK53994.1 Pakistan
GWHPAOJI000001 Pakistan-Punjab
GWHPAOLE000001 Pakistan-KPK
GWHPAOJF000001 Pakistan-KPK
GWHPAOJN000001 Pakistan-Capital
GWHPAOLN000001 Pakistan-Punjab
GWHPAOJG000001 Pakistan-Punjab
GWHPAOMY000001 Pakistan-Capital
QHR63299.1-BatCoV-RaTG13
AVP78030.1-Bat-SARS-like-CoV
AVP78041.1-Bat-SARS-like-CoV
AID16715.1-Bat-SARS-like-CoV
AIA62309.1-BtRs-BetaCoV-2014
AAR87543.1-SARS-CoV-TW4-2003
ADC35510.1-SARS-CoV-HKU39849
AAR87587.1-SARS-CoV-TW8-2003
NP 828849.2-SARS-CoV-2003
AAP33696.1-SARS-CoV-2003
ACZ71766.1-SARS-CoV-wticMB
AAP13442.1-SARS-CoV-Urbani-200
AAP13566.1-SARS-CoV-CUHK-W1
AAP30028.1-SARS-CoV-BJ01-2003
AAV91630.1-SARS-CoV-A022-civet
AAS00002.1-SARS-Cov-GZ02-2003
ATO98143.1-Bat-SARS-like-CoV
ATO98179.1-Bat-SARS-like-CoV
ATO98155.1-Bat-SARS-like-CoV
ATO98191.1-Bat-SARS-like-CoV
ARI44803.1-Bat-CoV-2020
QDF43824.1-BtRs-BetaCoV-2018
QDF43834.1-BtRs-BetaCoV-2018
QDF43829.1-BtRs-BetaCoV-2018
AGZ48830.1-Bat-SARS-likeCoV-W1V1
ATO98216.1-Bat-SARS-like-CoV
ATO98130.1-Bat-SARS-like-CoV
AGZ48805.1-Bat-SARS-like-CoV
YP 009072438.1-Bat-betaCoV/Zhejiang2013
YP 009047202.1-MERS-CoV-2014
AIY60526.1-MERS-CoV-2014
ASU90338.1-MERS-CoV-2017
AGV08377.1-MERS-CoV-2013
ALR69640.1-MERS-CoV-2015
AHB33324.1-MERS-CoV-2013
ALX27230.1-MERS-CoV-2015
ASU90679.1-MERS-CoV-2017
AHX71944.1-MERS-CoV-2014
AGN72639.1-MERS-CoV-2013
YP 009555238.1-Human-CoV-OC43
AAR01012.1-Human-CoV-OC43-2003
AGT51508.1-Human-CoV-OC43-2013
AXX83348.1-Human-CoV-OC43-2017
AEN19363.1-Human-CoV-OC43-2011
AXX83342.1-Human-CoV-OC43-2017
Betacoronavirus
QCX35177.1-Rhinolophus-bat-CoV
AFU92130.1-Hipposideros-bat-CoV
ALA50248.1-Camel-alphaCoV-2015
ATN23888.1-Rhinolophus-bat-CoV
AWJ64262.1-Porcine-enteric-alphaCoV
YP 001552234.1-Rhinolophus-bat-CoV
ABQ57215.1-Rhinolophus-bat-CoV
ABQ57223.1-Rhinolophus-bat-CoV
Alphacoronavirus
QDQ69145.1-Infectious-bronchitis-virus
QDQ69119.1-Infectious-bronchitis-virus
CAZ86697.1-Infectious-bronchitis-virus
QGM12427.1-Infectious-bronchitis-virus
ADA83476.1-Infectious-bronchitis-virus
ADV71823.1-Infectious-bronchitis-virus
ADV71773.1-Infectious-bronchitis-virus
Gammacoronavirus
YP 005352853.1-Magpie-robinCoV
AXP20280.1-Quail-delta-CoV
YP 005352845.1-Sparrow-CoV-HKU17
QDH76194.1-Porcine-deltaCoV
ASR75147.1-Porcine-deltaCoV
AKC54434.1-Porcine-delta-CoV
Deltacoronavirs
0.050

Fig. S1. Neighbor Joining tree of SARS-CoV-2 isolates from Pakistan based on the 7,096-aa polyprotein pp1ab. Phylogenetic analysis involved 122 pp1ab sequences from the subfamily Coronavirinae, including representatives of four genera; α -CoV, β-CoV ,γ-CoV, and δ-CoV. The phylogenetic tree was reconstructed using the neighbor Joining (NJ) method with p-distance amino acid substitution model. Bootstrap values>50% are shown along the branches. Scale bar shows amino acid substitution per site. Tree branches representingSARS-CoV-2 isolates from Pakistan are shown in red color. The other Coronavirinae species were color coded as Alphacoronavirus; fuchsia, Betacoronavirus; olive Bat-CoV, lime Bat-SL-CoV, blue BtRs-Beta-CoV, black SARS-CoV,purple MERS-CoV, aqua Human-CoV-OC43, Gammacoronavirus ;silver and Deltacoronavirus; teal.
