# Supplementary material for: Unraveling the genetic variations underlying virulence disparities among SARS-CoV-2 strains across global regions: insights from Pakistan
Source: Virol J. 2024 Mar 6;21:55. doi: 10.1186/s12985-024-02328-8 (PMC10916261; doi:10.1186/s12985-024-02328-8)
Supplement: Supplementary file 2 — Supplementary Material 2 [file 12985_2024_2328_MOESM2_ESM.pdf]

Supplementary data for:

Unraveling the Genetic Variations Underlying Virulence Disparities Among SARS-CoV-2 Strains: Insights from Pakistan

Momina Jabeen[a], Shifa Shoukat[a], Huma Shireen[a], Yiming Bao[b, c], Abbas Khan[d,e], & Amir Ali Abbasi[a]*

[a]National Center for Bioinformatics, Program of Comparative and Evolutionary Genomics, Faculty of Biological Sciences, Quaid-i-Azam University, Islamabad 45320, Pakistan

[b]National Genomics Data Center & CAS Key Laboratory of Genome Sciences and Information, Beijing Institute of Genomics, Chinese Academy of Sciences, and China National Center for Bioinformation, Beijing 100101, China

[c]University of Chinese Academy of Sciences, Beijing 100101, China

[d]Department of Bioinformatics and Biological Statistics, School of Life Sciences and Biotechnology, Shanghai Jiao Tong University, Shanghai 200240, China.

[e]School of Medical and Life Sciences, Sunway University, Sunway City, Malaysia.

*Corresponding author (A.A.A) Email: abbasiam@qau.edu.pk

Email Addresses

MJ: E-mail: mominajabeen999@gmail.com

SS: E-mail:shifabinteshoukat@gmail.com

HS: E-mail: humashireen.qau@gmail.com

YB: E-mail: baoym@big.ac.cn

AK: E-mail: abbaskhan@sjtu.edu.cn

AAA*: E-mail:abbasiam@qau.edu.pk

Table S1. The pp1ab polyprotein sequences derived from the genomes of SARS-CoV-2 sampled from different locations of Pakistan during first pandemic wave (March 01, 2020, to June 30, 2020).								
**S.No**	**Protein accession (NCBI)**	**Protein accession (2019-nCoVR)**	**Protein accession (GISAID)**	**Protein Length (aa)**	**Collection Date**	**Pangolin Lineage**	**Locality**	**Clinical Symptoms**
1.	QQH15664	GWHPAOJE000001	-	7096	2020-05-28	B.1.605	Punjab	Fever, Sore Throat
2.	QQH15676	GWHPAOJF000001	-	7096	2020-05-28	B.1	KPK	Asymptomatic
3.	QQH15688	GWHPAOJG000001	-	7096	2020-05-28	B.1	Punjab	Fever, Cough, Body Pain, No Smell and Taste, Hypertension
4.	QQH15700	GWHPAOJH000001	-	7096	2020-05-28	B.1	Punjab	Fever, Blood Pressure, Diabetes
5.	QQH15712	GWHPAOJI000001	-	7096	2020-05-29	B.4	Punjab	Asymptomatic
6.	QQH15724	GWHPAOJJ000001	-	7096	2020-05-29	B.1	Punjab	Asymptomatic
7.	QQH15736	GWHPAOJK000001	-	7096	2020-05-29	B.6	Federal Capital	Asymptomatic
8.	QQH15748	GWHPAOJL000001	-	7096	2020-05-31	B.1	Federal Capital	Asymptomatic
9.	QQH15760	GWHPAOJM000001	-	7096	2020-05-31	B.1	Federal Capital	Asymptomatic
10.	QQH15772	GWHPAOJN000001	-	7096	2020-05-31	B.1	Federal Capital	Fever
11.	QQH15784	GWHPAOJO000001	-	7096	2020-05-31	B.1	Federal Capital	Asymptomatic
12.	QQH15796	GWHPAOJP000001	-	7096	2020-05-29	B.1.36.10	Punjab	Fever, Cough, Myalgia
13.	QQH15808	GWHPAOJQ000001	-	7096	2020-05-30	B.1.1.303	Federal Capital	Asymptomatic
14.	QQH15820	GWHPAOJR000001	-	7096	2020-05-30	B.1	Federal Capital	Asymptomatic
15.	QQH15832	GWHPAOJS000001	-	7096	2020-05-29	B.1.36.24	Federal Capital	Asymptomatic
16.	QQH15844	GWHPAOJT000001	-	7096	2020-05-29	B.1.36.24	Federal Capital	Asymptomatic
17.	QQH15856	GWHPAOJU000001	-	7096	2020-05-30	B.1	Federal Capital	Abdomen Pain
18.	QQH15868	GWHPAOJV000001	-	7096	2020-05-30	B.1.1.1	Federal Capital	Fever
19.	QQH15880	GWHPAOJW000001	-	7096	2020-05-30	A	Federal Capital	Fever, Cough
20.	QQH15892	GWHPAOJX000001	-	7096	2020-05-30	B.1.1	Federal Capital	Fever, Cough, Sore Throat
21.	QQH15904	GWHPAOJY000001	-	7096	2020-05-30	B.1.1.1	Federal Capital	Asymptomatic
22.	QQH15916	GWHPAOJZ000001	-	7096	2020-05-29	B.1	Federal Capital	Fever, Cough, SOB

23.	QQH15928	GWHPAOKA000001	-	7096	2020-05-29	B.1.1	Federal Capital	Fever, Cough, SOB
24.	QQH15940	GWHPAOKB000001	-	7096	2020-05-30	B.1.1	Punjab	Asymptomatic
25.	QQH15952	GWHPAOKC000001	-	7096	2020-05-30	B.1.1.1	Federal Capital	Asymptomatic
26.	QQH15964	GWHPAOKD000001	-	7096	2020-05-31	B.1	Punjab	Asymptomatic
27.	QQH15976	GWHPAOKE000001	-	7096	2020-05-30	B.1	Federal Capital	Fever, Body Pain
28.	QQH15988	GWHPAOKF000001	-	7096	2020-05-29	B.1	Federal Capital	Fever, Cough
29.	QQH16000	GWHPAOKG000001	-	7096	2020-05-29	B.1	Federal Capital	Asymptomatic
30.	QQH16012	GWHPAOKH000001	-	7096	2020-05-29	B.1	Federal Capital	Asymptomatic
31.	QQH16024	GWHPAOKI000001	-	7096	2020-05-29	B.6	Federal Capital	Asymptomatic
32.	QQH16036	GWHPAOKJ000001	-	7096	2019-11-30	B.1	Punjab	Sore Throat
33.	QQH16048	GWHPAOKK000001	-	7096	2020-05-29	B.1	Federal Capital	Asymptomatic
34.	QQH16060	GWHPAOKL000001	-	7096	2020-05-29	B.1	Federal Capital	Asymptomatic
35.	QQH16072	GWHPAOKM000001	-	7096	2020-05-29	B.1.1.1	Federal Capital	Fever, Sore Throat, Cough
36.	QQH16084	GWHPAOKN000001	-	7096	2020-05-29	B.1.605	Federal Capital	Fever, Sore Throat
37.	QQH16096	GWHPAOKO000001	-	7096	2020-05-29	B.1	Federal Capital	Fever, Sore Throat
38.	QQH16108	GWHPAOKP000001	-	7096	2020-05-29	B.1.36.36	Federal Capital	Asymptomatic
39.	QQH16120	GWHPAOKQ000001	-	7096	2020-05-29	B.1	Punjab	Asymptomatic
40.	QQH16132	GWHPAOKR000001	-	7096	2020-05-29	B.1.605	Federal Capital	Asymptomatic
41.	QQH16144	GWHPAOKS000001	-	7096	2020-05-29	B.1	Federal Capital	Fever, Cough
42.	QQH16156	GWHPAOKT000001	-	7096	2020-05-29	B.1.36.36	Federal Capital	SOB, Hospital Admission
43.	QQH16168	GWHPAOKU000001	-	7096	2020-05-29	B.1.605	Federal Capital	Fever, Cough
44.	QQH16180	GWHPAOKV000001	-	7096	2020-05-29	B.1.605	Punjab	Fever, Cough
45.	QQH16192	GWHPAOKW000001	-	7096	2020-05-30	B.1.605	Federal Capital	Asymptomatic
46.	QQH16204	GWHPAOKX000001	-	7096	2020-05-30	B.1.605	Federal Capital	Fever
47.	QQH16216	GWHPAOKY000001	-	7096	2020-05-30	B.1	Federal Capital	Cough, Fever, Sore Throat, Fatigue
48.	QQH16228	GWHPAOKZ000001	-	7096	2020-05-29	B.1.605	Federal Capital	Asymptomatic
49.	QQH16240	GWHPAOLA000001	-	7096	2020-05-30	B.1	Punjab	Asymptomatic

50.	QQH16252	GWHPAOLB000001	-	7094	2020-05-30	B.6	Punjab	-
51.	QQH16264	GWHPAOLC000001	-	7096	2019-11-30	B.1.36	Punjab	Asymptomatic
52.	QQH16276	GWHPAOLD000001	-	7096	2020-05-30	B.1.1	KPK	Asymptomatic
53.	QQH16288	GWHPAOLE000001	-	7096	2020-05-30	B.1	KPK	Symptomatic
54.	QQH16300	GWHPAOLF000001	-	7096	2020-05-30	B.1	Federal Capital	Asymptomatic
55.	QQH16312	GWHPAOLG000001	-	7096	2020-05-30	B.1	Federal Capital	Asymptomatic
56.	QQH16324	GWHPAOLH000001	-	7096	2020-05-30	B.1.1	Federal Capital	Fever, Vomiting
57.	QQH16336	GWHPAOLI000001	-	7096	2020-05-30	B.1	Punjab	Fever
58.	QQH16348	GWHPAOLJ000001	-	7096	2020-05-30	B.1.36	Punjab	Asymptomatic
59.	QQH16360	GWHPAOLK000001	-	7096	2020-05-30	B.1.1.1	Punjab	Asymptomatic
60.	QQH16372	GWHPAOLL000001	-	7096	2020-05-30	B.1.1.1	Punjab	Asymptomatic
61.	QQH16384	GWHPAOLM000001	-	7096	2020-05-30	B.1	Punjab	Asymptomatic
62.	QQH16396	GWHPAOLN000001	-	7096	2020-05-30	B.1.36.24	Punjab	Asymptomatic
63.	QQH16408	GWHPAOLO000001	-	7094	2020-05-30	B.1	Punjab	Asymptomatic
64.	QQH16420	GWHPAOLP000001	-	7096	2020-05-30	B.1	Punjab	Asymptomatic
65.	QQH16432	GWHPAOLQ000001	-	7096	2020-05-30	B.1.36	Punjab	Asymptomatic
66.	QQH16444	GWHPAOLR000001	-	7096	2020-05-30	B.1	Punjab	Asymptomatic
67.	QQH16456	GWHPAOLS000001	-	7096	2020-05-30	B.1	Punjab	Asymptomatic
68.	QQH16468	GWHPAOLT000001	-	7096	2020-05-30	B.1	Punjab	Asymptomatic
69.	QQH16480	GWHPAOLU000001	-	7096	2020-05-30	B.1	Punjab	Asymptomatic
70.	QQH16492	GWHPAOLV000001	-	7096	2020-06-01	B.1.1.1	Federal Capital	Cough
71.	QQH16504	GWHPAOLW000001	-	7096	2020-06-01	B.1	Federal Capital	Asymptomatic
72.	QQH16516	GWHPAOLX000001	-	7096	2019-11-30	B.1	Punjab	Cough, Body pain, Sore Throat
73.	QQH16528	GWHPAOLY000001	-	7096	2020-05-30	B.1	Punjab	Asymptomatic
74.	QQH16540	GWHPAOLZ000001	-	7096	2020-05-31	B.1.1.1	Federal Capital	Fever, Sore Throat, Cough
75.	QQH16552	GWHPAOMA000001	-	7096	2020-05-31	B.1	Federal Capital	Fever, Cough
76.	QQH16564	GWHPAOMB000001	-	7096	2020-05-31	B.1	Punjab	Asymptomatic

77.	QQH16576	GWHPAOMC000001	-	7096	2020-05-31	B.1.1	Punjab	Fever
78.	QQH16588	GWHPAOMD000001	-	7096	2020-05-31	B.1	Punjab	Asymptomatic
79.	QQH16600	GWHPAOME000001	-	7096	2020-05-31	B.1	Punjab	Asymptomatic
80.	QQH16612	GWHPAOMF000001	-	7096	2020-05-31	B.1	Punjab	Fever, SOB, Body Pain, No Smell
81.	QQH16624	GWHPAOMG000001	-	7096	2020-05-31	B.1	Punjab	Asymptomatic
82.	QQH16636	GWHPAOMH000001	-	7096	2020-05-31	B.1	Punjab	Asymptomatic
83.	QQH16648	GWHPAOMI000001	-	7096	2020-04-24	B.1	Federal Capital	Fever
84.	QQH16660	GWHPAOMJ000001	-	7096	2020-04-29	B.1	Punjab	Fever
85.	QQH16672	GWHPAOMK000001	-	7096	2020-04-16	B.1.1.1	Gilgit Baltistan	Asymptomatic
86.	QQH16684	GWHPAOML000001	-	7096	2020-04-16	B.1.1.1	Gilgit Baltistan	Fever, Cough
87.	QQH16696	GWHPAOMM000001	-	7096	2020-04-25	B.1	Federal Capital	Fever, Cough
88.	QQH16708	GWHPAOMN000001	-	7096	2020-04-25	B.1.1.1	Federal Capital	Asymptomatic
89.	QQH16720	GWHPAOMO000001	-	7096	2020-04-15	B.1.1.1	KPK	Asymptomatic
90.	QQH16732	GWHPAOMP000001	-	7096	2020-04-15	B.1.1.1	KPK	Asymptomatic
91.	QQH16744	GWHPAOMQ000001	-	7096	2020-04-15	B.1	KPK	Asymptomatic
92.	QQH16756	GWHPAOMR000001	-	7096	2020-04-15	B.1	KPK	Asymptomatic
93.	QQH16768	GWHPAOMS000001	-	7096	2020-04-15	B.1	KPK	Fever, SOB, Cough
94.	QQH16780	GWHPAOMT000001	-	7096	2020-04-15	B.1	KPK	Asymptomatic
95.	QQH16792	GWHPAOMU000001	-	7096	2020-04-22	B.1	Federal Capital	Fever
96.	QQH16804	GWHPAOMV000001	-	7096	2020-04-15	B.1	KPK	Asymptomatic
97.	QQH16816	GWHPAOMW000001	-	7096	2020-04-22	B.1	-	Asymptomatic
98.	QQH16828	GWHPAOMX000001	-	7096	2020-04-25	B.1.36.36	Federal Capital	Fever
99.	QQH16840	GWHPAOMY000001	-	7096	2020-05-01	B.1	Federal Capital	Asymptomatic
100.	QQH16852	GWHPAOMZ000001	-	7096	2020-04-15	B.1	KPK	Asymptomatic
101.	QQH16864	GWHPAONA000001	-	7096	2020-04-24	B.6	Federal Capital	Asymptomatic
102.	QQH16876	GWHPAONB000001	-	7096	2020-05-13	B.1	-	Asymptomatic
103.	QQH16888	GWHPAONC000001	-	7096	2020-04-22	B.1.36.36	Punjab	Asymptomatic

104.	QQH16900	GWHPAOND000001	-	7096	2020-04-24	B.1	Punjab	Asymptomatic
105.	QQH16912	GWHPAONE000001	-	7096	2020-04-30	B.6	Punjab	Cough, SOB, Sore Throat
106.	QQH16924	GWHPAONF000001	-	7096	2020-04-30	B.1	Punjab	Asymptomatic
107.	QQH16936	GWHPAONG000001	-	7096	2020-04-13	B.6	KPK	Asymptomatic, Cough, Sore Throat
108.	QQH16948	GWHPAONH000001	-	7096	2020-04-16	B.6	Federal Capital	Diabetes, Hypertension, Typhoid, Diarrhea
109.	QQH16960	GWHPAONI000001		7096	2020-05-11	B.1	Federal Capital	Asymptomatic
110.	QQH16972	GWHPAONJ000001	-	7096	2020-04-26	B.1	Punjab	Fever, SOB, Cough
111.	QQH16984	GWHPAONK000001	-	7096	2020-04-30	B.1	Punjab	Asymptomatic
112.	QQH16996	GWHPAONL000001	-	7096	2020-04-18	B.1	Punjab	Fever, Cough
113.	QQH17008	GWHPAONM000001	-	7096	2020-04-15	B.1.111	KPK	Asymptomatic
114.	QQH17020	GWHPAONN000001	-	7096	2020-04-30	B.1	Punjab	Asymptomatic
115.	QQH17032	GWHPAONO000001	-	7096	2020-04-20	B.1	Punjab	Asymptomatic
116.	QQH17044	GWHPAONP000001	-	7096	2020-05-12	B.1	Federal Capital	Fever, Cough
117.	QQH17056	GWHPAONQ000001	-	7096	2020-04-23	B.1	Federal Capital	Asymptomatic
118.	QQH17068	GWHPAONR000001	-	7096	2020-04-25	B.6	Federal Capital	Asymptomatic
119.	QQH17080	GWHPAONS000001	-	7096	2020-04-25	B.6	Federal Capital	Asymptomatic
120.	QQH17092	GWHPAONT000001	-	7096	2020-04-22	B.1	Punjab	Asymptomatic
121.	QQH17104	GWHPAONU000001	-	7096	2020-04-18	B.1	Federal Capital	Fever, Sore Throat, Body Ache
122.	QQH17116	GWHPAONV000001	-	7096	2020-04-30	B.1	Punjab	Asymptomatic
123.	QQH17128	GWHPAONW000001	-	7096	2020-04-22	B.1.605	Punjab	Asymptomatic
124.	QQH17140	GWHPAONX000001	-	7096	2020-04-22	B.1	Punjab	Asymptomatic
125.	QQH17152	GWHPAONY000001	-	7096	2020-04-16	B.1	Punjab	Diabetes, Hypertension, IHD
126.	QQH17164	GWHPAONZ000001	-	7096	2020-03-23	B.1.605	Punjab	Asymptomatic
127.	QQH17176	GWHPAOOA000001	-	7096	2020-03-25	B.1.36.36	Punjab	Asymptomatic
128.	QQH17188	GWHPAOOB000001	-	7096	2020-03-25	B.1	Punjab	Asymptomatic

129.	QQH17200	GWHPAOOC000001	-	7096	2020-03-22	B.1.1.1	Punjab	Fever, Diabetes
130.	QQH17212	GWHPAOOD000001	-	7096	2020-03-18	B.1.1	Punjab	Asymptomatic
131.	QQH17224	GWHPAOOE000001	-	7096	2020-03-30	B.1.1	Punjab	Asymptomatic
132.	QQH17236	GWHPAOOF000001	-	7096	2020-03-22	B.1	Punjab	Asymptomatic
133.	QQH17248	GWHPAOOG000001	-	7096	2020-03-22	B.1	Punjab	Asymptomatic
134.	QQH17260	GWHPAOOH000001	-	7096	2020-03-16	B.1	Federal Capital	Fever
135.	QQH17272	GWHPAOOI000001	-	7096	2020-03-16	B.1.1.1	Federal Capital	Asymptomatic
136.	QQH17284	GWHPAOOJ000001	-	7096	2020-03-16	B.1	Federal Capital	Asymptomatic
137.	QQH17296	GWHPAOOK000001	-	7096	2020-03-16	B.1.1	Baluchistan	Asymptomatic
138.	QQH17308	GWHPAOOL000001	-	7096	2020-03-16	B	Baluchistan	Asymptomatic
139.	QQH17320	GWHPAOOM000001	-	7096	2020-05-30	B.1	Federal Capital	Asymptomatic
140.	QQH17332	GWHPAOON000001	-	7096	2020-05-30	B.1	Federal Capital	Asymptomatic
141.	QQH17344	GWHPAOOO000001	-	7096	2020-05-30	B.1.1.1	Federal Capital	Asymptomatic
142.	QQH17356	GWHPAOOP000001	-	7096	2020-05-30	B.1.1.303	Federal Capital	Fever, Cough
143.	QQH17368	GWHPAOOQ000001	-	7096	2020-05-30	B.1	Federal Capital	Asymptomatic
144.	QQH17380	GWHPAOOR000001	-	7096	2020-05-30	B.1	Federal Capital	Asymptomatic
145.	QQH17392	GWHPAOOS000001	-	7096	2020-05-30	B.1	Federal Capital	Asymptomatic
146.	QQH17404	GWHPAOOT000001	-	7096	2020-05-30	B.1.1.1	Federal Capital	Fever, Cough, Flu, Body pain, No Smell and Taste
147.	QQH17416	GWHPAOOU000001	-	7096	2020-05-30	B.1.1.1	Federal Capital	Asymptomatic
148.	QQH17428	GWHPAOOV000001	-	7096	2020-05-30	B.1	Federal Capital	Asymptomatic
149.	QQH17440	GWHPAOOW000001	-	7096	2020-06-01	B.1	Punjab	Asymptomatic
150.	QQH17452	GWHPAOOX000001	-	7096	2020-06-01	B.1.36.36	Punjab	Asymptomatic
151.	QLD99663	-	EPI_ISL_2629942	7096	2020-05-20	B.1	Pakistan	-
152.	QLD99985	-	EPI_ISL_2629943	7096	2020-05-10	B.6	Pakistan	-
153.	QQK33666	-	-	7096	2020-06-24	B.1.36	Pakistan	-
154.	QQK53886	-	-	7096	2020-06-24	B.1	Pakistan	-

155.	QQK53898	-	-	7096	2020-05-29	B.1.471	Pakistan	-
156.	QQK53970	-	-	7096	2020-05-3	B.6	Pakistan	-
157.	QQK53982	-	-	7096	2020-03-16	B.4	Pakistan	-
158.	QQK53994	-	-	7096	2020-03-16	B.4	Pakistan	-
159.	QQS74323	-	-	7096	2020-06-24	B.1.471	Pakistan	-
160.	QLD99687	-	-	7096	2020-03-19	B.1	Pakistan	-
161.	QLD99699	-	-	7096	2020-03-19	B.1	Pakistan	-
162.	QLD99711	-	-	7096	2020-03-20	A	Pakistan	-
163.	QLD99723	-	-	7096	2020-03-19	A	Pakistan	-
164.	QQD86525	-	-	7096	2020-06-10	B.1	Pakistan	-
165.	QQL13872	-	EPI_ISL_779255	7096	2020-05-20	B.1	Karachi	-
166.	QQL13884	-	EPI_ISL_779256	7096	2020-05-20	B.1.471	Karachi	-
167.	QQL13896	-	EPI_ISL_779257	7096	2020-05-3	B.1	Karachi	-
168.	QQL13908	-	EPI_ISL_779258	7096	2020-05-20	B.1.471	Karachi	-
169.	QQL13931	-	EPI_ISL_779260	7096	2020-06-02	B.1.36	Karachi	-
170.	QQL13954	-	EPI_ISL_779262	7096	2020-06-02	B.1.36	Karachi	-
171.	QQL13977	-	EPI_ISL_779264	7096	2020-06-03	A	Karachi	-
172.	QQL13989	-	EPI_ISL_779265	7096	2020-06-02	B.1.36	Karachi	-
173.	QQL14001	-	EPI_ISL_779266	7096	2020-06-02	B.1.471	Karachi	-
174.	QQL14013	-	EPI_ISL_779267	7096	2020-06-02	B.1.471	Karachi	-
175.	QQL14025	-	EPI_ISL_779268	7096	2020-06-03	B.1	Karachi	-
176.	QQL14037	-	-	7096	2020-06-04	B.6	Karachi	-
177.	QQL14049	-	EPI_ISL_779269	7096	2020-06-04	B.1.36	Karachi	-
178.	QQL14073	-	EPI_ISL_779271	7096	2020-06-14	B.1.471	Karachi	-
179.	QQL14166	-	EPI_ISL_779279	7096	2020-06-14	B.1.471	Karachi	-
180.	QQL14178	-	EPI_ISL_779280	7096	2020-06-14	B.1	Karachi	-
181.	QQL14274	-	EPI_ISL_779288	7096	2020-06-14	B.1.471	Karachi	-
182.	QPB18038	-	EPI_ISL_632908	7096	2020-06-2	B.6	Pakistan	-

183.	QNV71166	-	EPI_ISL_548942	7096	2020-05-11	C.23	Pakistan	-
184.	QNV71178	-	EPI_ISL_548943	7096	2020-05-11	C.23	Pakistan	-
185.	QNV71190	-	EPI_ISL_548944	7096	2020-05-11	C.23	Pakistan	-
186.	QNV71202	-	EPI_ISL_548945	7096	2020-05-11	C.23	Pakistan	-
187.	QNV71214	-	EPI_ISL_548946	7096	2020-05-11	B.1.1	Pakistan	-
188.	QNC49338	-	EPI_ISL_513925	7096	2020-05-15	B.1	Pakistan	-
189.	QJX57851	-	EPI_ISL_451958	7095	2020-03-16	B	Karachi	-
190.	QIS60274	-	-	7084	2020-03-12	B	KPK	-
191.	QIQ22758	-	EPI_ISL_417444	7096	2020-03-04	B.4	Gilgit	-
192.	-	-	EPI_ISL_1385796	-	2020-06-06	B.1.36	Islamabad	-
193.	-	-	EPI_ISL_1385798	-	2020-06-06	B.1	Islamabad	-
194.	-	-	EPI_ISL_1406395	-	2020-06-06	B.1	Islamabad	-
195.	-	-	EPI_ISL_1406397	-	2020-06-06	B.1	Islamabad	-
196.	-	-	EPI_ISL_1406400	-	2020-06-06	B.1	Islamabad	-
197.	-	-	EPI_ISL_468159	-	2020-06-02	A	Islamabad	-
198.	-	-	EPI_ISL_468160	-	2020-06-02	B.1	Rawalpindi	-
199.	-	-	EPI_ISL_468161	-	2020-06-02	B.1	Islamabad	-
200.	-	-	EPI_ISL_468162	-	2020-06-02	B.1	Islamabad	-
201.	-	-	EPI_ISL_468163	-	2020-06-02	A	Islamabad	-
202.	-	-	EPI_ISL_708839	-	2020-06-06	B.1.36	Karachi	-
203.	-	-	EPI_ISL_708840	-	2020-06-10	B.1.36	Karachi	-

This table provides the accession numbers of pp1ab polyprotein, their respective amino acid (aa) length, sample collection date, **Pangolin** lineage, and source locality. The clinical symptoms for subset of samples are also given. Please note; the data presented in this table is derived from all available completely sequenced genomes of SARS-CoV-2 sampled from the Pakistani population during the first wave of the pandemic (March 01, 2020 to June 30, 2020).

Table S2. The pp1ab polyprotein sequences from the subfamily Coronavirinae, including representatives of four genera: Alphacoronavirus, Betacoronavirus, Gammacoronavirus, and Deltacoronavirus					
**S.No**	**Protein accession (NCBI)**	**Protein length (aa)**	**Name**	**Genera**	**Year**
1	NP_828849.2	7073	SARS-CoV	Betacoronavirus	2003
2	YP_009047202.1	7078	MERS-CoV	Betacoronavirus	2012
3	YP_009072438.1	7247	Bat Hp-beta-CoV/ Zhejiang2013	Betacoronavirus	2013
4	ATO98143.1	7073	Bat SARS-like-CoV	Betacoronavirus	2016
5	AAS00002.1	7073	SARS-Cov GZ02	Betacoronavirus	2003
6	QDF43824.1	7073	BtRs-BetaCoV/YN2018B	Betacoronavirus	2018
7	AIA62309.1	7070	BtRs-BetaCoV/HuB2013	Betacoronavirus	2014
8	AAV91630.1	7073	SARS-CoV A022	Betacoronavirus	2004
9	QHR63299.1	7095	BatCoV RaTG13	Betacoronavirus	2020
10	AVP78030.1	7092	Bat SARS-like CoV (bat-SL-CoVZC45)	Betacoronavirus	2018
11	AID16715.1	7063	Bat SARS-like CoV	Betacoronavirus	2013
12	AGZ48830.1	7073	Bat SARS-like CoV WIV1	Betacoronavirus	2013
13	AAP13566.1	7073	SARS CoV CUHK-W1	Betacoronavirus	2003
14	ACZ71766.1	7073	SARS CoV wtic-MB	Betacoronavirus	2009
15	AAP13442.1	7073	SARS CoV Urbani	Betacoronavirus	2003
16	AAR87543.1	7073	SARS CoV TW4	Betacoronavirus	2003
17	ADC35510.1	7073	SARS CoV HKU-39849	Betacoronavirus	2010
18	AAP33696.1	7073	SARS CoV	Betacoronavirus	2003
19	ATO98130.1	7073	Bat SARS-like CoV	Betacoronavirus	2016
20	AGZ48805.1	7073	Bat SARS-like CoV RsSHC014	Betacoronavirus	2013
21	AAP30028.1	7073	SARS CoV BJ01	Betacoronavirus	2003

22	AAR87587.1	7073	SARS CoV TW8	Betacoronavirus	2003
23	ATO98216.1	7073	Bat SARS-like CoV	Betacoronavirus	2016
24	AVP78041.1	7070	Bat SARS-like CoV (bat-SL-CoVZXC21)	Betacoronavirus	2018
25	ARI44803.1	7073	Bat CoV	Betacoronavirus	2017
26	ATO98191.1	7073	Bat SARS-like CoV	Betacoronavirus	2016
27	QDF43834.1	7073	CoV BtRs-BetaCoV/YN2018D	Betacoronavirus	2018
28	QDF43829.1	7073	CoV BtRs-BetaCoV/YN2018C	Betacoronavirus	2018
29	ATO98155.1	7073	Bat SARS-like CoV	Betacoronavirus	2016
30	ATO98179.1	7073	Bat SARS-like CoV	Betacoronavirus	2016
31	AGV08377.1	7078	MERS-CoV	Betacoronavirus	2013
32	ASU90679.1	7078	MERS-CoV	Betacoronavirus	2017
33	AHX71944.1	7078	MERS-CoV	Betacoronavirus	2014
34	AGN72639.1	7078	MERS-CoV	Betacoronavirus	2013
35	ALR69640.1	7078	MERS-CoV	Betacoronavirus	2015
36	AIY60526.1	7078	MERS-CoV	Betacoronavirus	2014
37	ASU90338.1	7078	MERS-CoV	Betacoronavirus	2017
38	ALX27230.1	7078	MERS-CoV	Betacoronavirus	2015
39	AHB33324.1	7078	MERS-CoV	Betacoronavirus	2013
40	YP_009555238.1	7095	Human CoV-OC43	Betacoronavirus	2019
41	AAR01012.1	7095	Human CoV-OC43	Betacoronavirus	2003
42	AGT51508.1	7095	Human CoV-OC43	Betacoronavirus	2013
43	AEN19363.1	7095	Human CoV-OC43	Betacoronavirus	2011
44	AXX83342.1	7095	Human CoV-OC43	Betacoronavirus	2017
45	AXX83348.1	7095	Human CoV-OC43	Betacoronavirus	2017
46	YP_001552234.1	6727	Rhinolophus bat CoV HKU2	Alphacoronavirus	2007
47	ABQ57215.1	6727	Rhinolophus bat CoV HKU2	Alphacoronavirus	2007

48	ABQ57223.1	6727	Rhinolophus bat CoV HKU2	Alphacoronavirus	2007
49	ATN23888.1	6728	Rhinolophus bat CoV HKU2	Alphacoronavirus	2017
50	AWJ64262.1	6728	Porcine enteric alphaCoV	Alphacoronavirus	2018
51	QCX35177.1	6712	Rhinolophus bat CoV HKU32	Alphacoronavirus	2019
52	AFU92130.1	6783	Hipposideros bat CoV HKU10	Alphacoronavirus	2012
53	ALA50248.1	6763	Camel alphaCoV	Alphacoronavirus	2015
54	ADV71823.1	6630	Infectious bronchitis virus	Gammacoronavirus	2010
55	ADV71773.1	6630	Infectious bronchitis virus	Gammacoronavirus	2010
56	ADA83476.1	6630	Infectious bronchitis virus-<br>Mass41	Gammacoronavirus	2009
57	CAZ86697.1	6631	Infectious bronchitis virus ITA/90254/2005	Gammacoronavirus	2009
58	QGM12427.1	6639	Infectious bronchitis virus	Gammacoronavirus	2019
59	QDQ69145.1	6627	Infectious bronchitis virus	Gammacoronavirus	2019
60	QDQ69119.1	6623	Infectious bronchitis virus	Gammacoronavirus	2019
61	ASR75147.1	6265	Porcine delta CoV	Coronavirinae	2017
62	AKC54434.1	6267	Porcine delta CoV	Deltacoronavirus	2015
63	QDH76194.1	6265	Porcine delta CoV	Deltacoronavirus	2019
64	YP_005352845.1	6277	Sparrow CoV HKU17	Deltacoronavirus	2011
65	AXP20280.1	6260	Quail delta CoV	Deltacoronavirus	2018
66	YP_005352853.1	6253	Magpie-robin CoV	Deltacoronavirus	2011

			HKU18		
67	YP_002308505.1	6307	Munia CoV HKU13-3514	Deltacoronavirus	2008

This table depicts the NCBI (National Center for Bioinformatics) derived accession numbers of pp1ab polyproteins, their amino acid (aa) length, defined name in literature and taxonomic classification. Dates of submission in NCBI are also given in the last column. Note: The list of corresponding homologous sequences from SARS-CoV-2 are given in Table S1.

**Table S3. The amino acid substitutions in pp1ab of SARS-CoV-2 (Pakistani isolates) in relation to the reference sequence for the Wuhan strain (YP_009724389.1) and closely related bat-CoVs.**							
**S.No**	**Amino Acid position**	**Bat-SL-CoV (AVP78030.1)**	**Bat-CoV-RaTG13 (QHR63299.1)**	**SARS-CoV-2 (YP_009724389.1)**	**Pak Isolates**	**Localization**	**Accession No**
1.	12	T	T	T	*	Nsp1	QQH16144
2.	16	L	L	L	H<br>*<br>*	Nsp1	QQH16144<br>QQH16108<br>QQH17176
3.	63	Q	Q	Q	L	Nsp1	QQL14274
4.	76	A	A	A	V	Nsp1	QQH16444
5.	82	G	G	G	S	Nsp1	QQL14025
6.	85	M	M	M	*<br>*	Nsp1	QQH16252<br>QQH16408
7.	86	V	V	V	*<br>*	Nsp1	QQH16252<br>QQH16408
8.	87	E	E	E	K	Nsp1	QQH16408
9.	112			G	C	Nsp1	EPI_ISL_1406395
10.	116	V	V	V	L	Nsp1	QQL14073
11.	170	T	T	T	*	Nsp1	QQH16312
12.	181	A	A	A	T	Nsp2	QQH17188
13.	196	Y	Y	Y	N	Nsp2	QQH16384
14.	207	R	R	R	C<br>C<br>C<br>C<br>C	Nsp2	QQH17284<br>QQH17440<br>QQK53982<br>QQK53994<br>QIQ22758
15.	212	S	S	S	L<br>L	Nsp2	QQH17416<br>QQH17404
16.	222	I	I	I	M<br>M<br>M	Nsp2	QQH15856<br>QQH16876<br>QQH16612
17.	224			T	I<br>I	Nsp2	EPI_ISL_1385798<br>EPI_ISL_1406400
18.	261	K	K	K	N<br>N	Nsp2	QQL14073<br>QQL14166
19.	265	T	T	T	*	Nsp2	QQH15748
20.	266	F	F	F	*	Nsp2	QQH17020
21.	275	F	F	F	L<br>L<br>L	Nsp2	QQL13931<br>QQL13954<br>QQL13989
22.	279	S	S	S	F<br>F	Nsp2	QQH16744<br>QQH16756
23.	287	R	R	R	G	Nsp2	QQL14178
24.	292	K	K	K	E	Nsp2	QPB18038
25.	302	S	S	S	F<br>F	Nsp2	QQH16672<br>QQH16720
26.	322	K	K	K	R	Nsp2	QQH16444
27.	378	V	V	V	I<br>I<br>I<br>I	Nsp2	QQH15712<br>QQK53982<br>QQK53994<br>QIQ22758
28.	391	S	S	S	F	Nsp2	QQH16492
29.	428	S	S	S	*	Nsp2	QQH17452

					G		QQH16336
30.	448	D	D	D	*	Nsp2	QJX57851
31.	450	L	L	L	*<br>*<br>*<br>*<br>*<br>*<br>*<br><br>F<br>F<br>F<br>F<br>F<br>F<br>F	Nsp2	QQH16120<br>QQH16084<br>QQH16132<br>QQH16180<br>QQH16120<br>QQH16108<br>QQH16096<br><br>QQH16924<br>QQH16804<br>QQH15916<br>QQH16060<br>QQH17428<br>EPI_ISL_1406397<br>EPI_ISL_468162
32.	505	S	S	S	F	Nsp2	QQH16576
33.	559	V	V	V	M	Nsp2	QQH16276
34.	586	F	F	F	L	Nsp2	QQH15964
35.	615	N	N	N	*	Nsp2	QQH17044
36.	627	V	V	V	F	Nsp2	QPB18038
37.	628	L	L	L	I	Nsp2	QQH17044
38.	687	A	A	A	V	Nsp2	QQH16000
39.	717	Y	Y	Y	*<br>*<br>*<br>*<br>*<br>*<br>*<br>*	Nsp2	QQH16144<br>QQH17104<br>QQH17128<br>QQH17164<br>QQH16240<br>QQH16204<br>QQH17296<br>QQH17044
40.	733	L	L	L	*	Nsp2	QQH15748
41.	748	P	P	P	L	Nsp2	QQH16552
42.	911	S	S	S	F	Nsp3	QQH16960
43.	913	E	E	E	G<br>G	Nsp3	QQH16456<br>QQH16468
44.	919	H	H	H	Y	Nsp3	QQH15988
45.	924	F	F	F	*	Nsp3	QQH15796
46.	934	D	G	G	C	Nsp3	QQL13908
47.	944	S	P	S	L<br>L<br>L<br>L	Nsp3	QNV71166<br>QNV71178<br>QNV71190<br>QNV71202
48.	999	E	T	T	I<br>I<br>I	Nsp3	QQL14001<br>EPI_ISL_1385798<br>EPI_ISL_1406400
49.	1016	S	P	L	F	Nsp3	QQK53970
50.	1022	T	T	T	I<br>I	Nsp3	QQK53982<br>QQK53994
51.	1083	M	M	M	I	Nsp3	EPI_ISL_1406395
52.	1125	G	G	G	C	Nsp3	QQH15880
53.	1175	L	L	L	I	Nsp3	QQL13872
54.	1246	T	T	T	I	Nsp3	QQH16492

					I<br>I<br>I<br>I<br>I<br>I<br>I<br>I<br>I<br>I<br>I<br>I<br>I<br>I<br>I<br>I<br>I<br>I<br>I<br>I<br>I		QQH16720<br>QQH17404<br>QQH17416<br>QQH17272<br>QQH16708<br>QQH16672<br>QQH16372<br>QQH15868<br>QQH15904<br>QQH16072<br>QQH16360<br>QQH16540<br>QQH16684<br>QQH16732<br>QQH17200<br>QQH17344<br>QQH15952<br>QNV71166<br>QNV71178<br>QNV71190<br>QNV71202
55.	1299	V	V	V	L<br>L	Nsp3	EPI_ISL_1385798<br>EPI_ISL_1406400
56.	1305	K	K	K	N<br>N<br>N<br>N	Nsp3	QNV71166<br>QNV71178<br>QNV71190<br>QNV71202
57.	1355	I	I	I	V<br>V	Nsp3	QQH17368<br>QQH17380
58.	1396	K	K	K	R	Nsp3	QQL14037
59.	1398	I	I	I	T	Nsp3	QQH17092
60.	1426	T	T	T	*	Nsp3	QQH16000
61.	1506	S	S	S	L	Nsp3	QQH16276
62.	1508	A	A	A	V<br>V<br>V	Nsp3	QQH17320<br>QQH17332<br>QQH15760
63.	1525	I	I	I	V	Nsp3	QQH16024
64.	1554	D	D	D	N	Nsp3	QQH16792
65.	1714	I	I	I	*	Nsp3	QQH17044
66.	1854	T	T	T	I<br>I	Nsp3	QQH16456<br>QQH16468
67.	1926	D	D	D	G	Nsp3	QQL14013
68.	2016	T	T	T	K<br>K<br>K<br>K<br>K<br>K<br>K<br>K<br><br>*<br>*<br>*<br>K	Nsp3	QQH16252<br>QQH16936<br>QQH16948<br>QQH17080<br>QQH16024<br>QQH16864<br>QQH16912<br>QQH17068<br><br>QQH15736<br>QQH15796<br>QQH17104<br>QLD99985

					K<br>K<br>K		QPB18038<br>QQK53970<br>QQL14037
69.	2030			S	L	Nsp3	EPI_ISL_1385796
70.	2133	V	V	V	A<br>A<br>A	Nsp3	QQL13931<br>QQL13954<br>QQL13989
71.	2259	M	M	M	I	Nsp3	QQH17080
72.	2274	T	T	T	I	Nsp3	QQH16792
73.	2497	K	K	K	N	Nsp3	QQH16792
74.	2511	K	K	K	N	Nsp3	QQH15928
75.	2564	L	L	L	F	Nsp3	QQL14025
76.	2608	K	K	K	*	Nsp3	QQH16144
77.	2648	T	T	T	I	Nsp3	QQH15964
78.	2696	A	A	A	*	Nsp3	QQH16240
79.	2702	Q	Q	Q	*<br>*<br>*<br>*<br>*<br>*<br>*<br>*<br>*<br>*<br>*<br>*<br>*<br>*<br>*<br>*<br>*<br>*<br>*<br><br>H<br>H<br>H<br>H<br>H<br>H<br>H<br>H<br>H<br>H<br>H<br>H<br>H<br>H<br>H<br>H<br>H<br>H<br>H<br>H	Nsp3	QQH17104<br>QQH17128<br>QQH17164<br>QQH16240<br>QQH17296<br>QQH17044<br>QQH16216<br>QQH16120<br>QQH16084<br>QQH16084<br>QQH16168<br>QQH16180<br>QQH16108<br>QQH16156<br>QQH16192<br>QQH15664<br>QQH15700<br>QQH17176<br>QQH16996<br><br>QQH16204<br>QQH16924<br>QQH16804<br>QQH15916<br>QQH16060<br>QQH17428<br>QQH16888<br>QQH17452<br>QQH16096<br>QQH16096<br>QQH15760<br>QQH17152<br>QQH17440<br>QQH17260<br>QQH17032<br>QQH16960<br>QQH16792<br>QQH16288<br>QQH15964<br>QQH17368

					H<br>H<br>H<br>H<br>H<br>H<br>H<br>H<br>H<br>H<br>H<br>H<br>H<br>H<br>H<br>H<br>H<br>H<br>H<br>H<br>H<br>H<br>H<br>H<br>H<br>H<br>H<br>H<br>H<br>H<br>H<br>H<br>H<br>H<br>H<br>H<br>H<br>H<br>H<br>H<br>H<br>H<br>H<br>H<br>H<br>H<br>H		QQH17380<br>QQH17320<br>QQH17332<br>QQH17188<br>QQH17116<br>QQH17092<br>QQH17056<br>QQH17020<br>QQH16840<br>QQH16816<br>QQH16768<br>QQH16564<br>QQH16552<br>QQH16516<br>QQH16384<br>QQH16336<br>QQH16312<br>QQH16012<br>QQH15676<br>QQH15724<br>QQH15772<br>QQH15784<br>QQH15820<br>QQH15976<br>QQH16528<br>QQH16588<br>QQH16624<br>QQH16636<br>QQH16648<br>QQH16660<br>QQH16900<br>QQH16972<br>QQH16984<br>QQH17140<br>QQH17236<br>QQH17248<br>QQH17284<br>QQH15688<br>QLD99663<br>QNC49338<br>QQL14178<br>QQL13896<br>EPI_ISL_1406397<br>EPI_ISL_468160<br>EPI_ISL_468161<br>EPI_ISL_468162
80.	2796	M	M	M	I<br>I<br>I	Nsp4	QQH15712<br>QQK53982<br>QQK53994
81.	2900	S	S	S	L	Nsp4	QQH15712
82.	2965	P	P	P	L	Nsp4	QIQ22758
83.	2981	S	S	S	*<br>*<br>*<br>*<br>*	Nsp4	QQH17104<br>QQH17128<br>QQH17164<br>QQH16888<br>QQH17116

84.	3018	F	F	F	L	Nsp4	QQH15916
85.	3040	A	A	A	S	Nsp4	QQH16372
86.	3058	T	T	T	I	Nsp4	QQH17272
87.	3072	G	G	G	C	Nsp4	QQH15748
88.	3099	S	S	S	*	Nsp4	QQH15736
89.	3153	F	F	F	-	Nsp4	QIS60274
90.	3154	Y	Y	Y	-	Nsp4	QIS60274
91.	3155	W	W	W	-	Nsp4	QIS60274
92.	3156	F	F	F	-	Nsp4	QIS60274
93.	3157	F	F	F	-	Nsp4	QIS60274
94.	3162	K	K	K	N	Nsp4	QQH16696
95.	3244	S	S	S	L<br>L	Nsp4	QQH16408<br>QQH16420
96.	3267	R	R	R	K	Nsp5	QQH15880
97.	3278	G	G	G	S<br>S<br>S<br>S<br>S<br>S<br>S<br>S<br>S<br>S<br>S<br>S<br>S<br>S<br>S<br>S<br>S<br>S<br>S<br>S<br>S<br>S	Nsp5	QQH16492<br>QQH16720<br>QQH17404<br>QQH17416<br>QQH17272<br>QQH16708<br>QQH16672<br>QQH16372<br>QQH15868<br>QQH15904<br>QQH16072<br>QQH16360<br>QQH16540<br>QQH16684<br>QQH16732<br>QQH17200<br>QQH17344<br>QQH15952<br>QNV71166<br>QNV71178<br>QNV71190<br>QNV71202
98.	3293	L	L	L	F	Nsp5	QQL14274
99.	3353	K	K	K	R	Nsp5	QQL13896
100.	3371	P	P	P	*	Nsp5	QQH15796
101.	3384	S	S	S	L<br>L	Nsp5	QQH16480<br>QQH16600
102.	3475	V	V	V	F	Nsp5	QQL14073
103.	3491			N	K	Nsp5	EPI_ISL_468160
104.	3492	D	D	D	*<br>*<br>*	Nsp5	QQH17104<br>QQH17128<br>QQH17164
105.	3497	A	A	A	V	Nsp5	QQH16924
106.	3603	F	F	F	*	Nsp6	QQH17032
107.	3606	V	V	L	F<br>F<br>F<br>F<br>F<br>F<br>F	Nsp6	QLD99985<br>QPB18038<br>QQK53970<br>QQL14037<br>QQK53982<br>QQK53994<br>QIQ22758

					F<br>F<br>F<br>F<br>F<br>F<br>F		QLD99663<br>QNC49338<br>QQL14178<br>EPI_ISL_1406397E<br>PI_ISL_468160<br>EPI_ISL_468161<br>EPI_ISL_468162
108.	3628	F	F	F	*	Nsp6	QQH16240
109.	3629	V	V	V	*	Nsp6	QQH16240
110.	3653	V	V	V	F	Nsp6	QQK33666
111.	3655	M	M	M<br>M	I<br>I<br>I	Nsp6	QQH15880<br>EPI_ISL_468159<br>EPI_ISL_468163
112.	3657	A	A	A	T	Nsp6	QQL14178
113.	3892	V	V	V	A	Nsp7	QQH17260
114.	3936	D	D	D	Y<br>Y	Nsp7	QQH16048<br>QQH16036
115.	4075	P	P	P	S	Nsp8	QQL14274
116.	4159	T	T	T	I<br>I	Nsp9	QQH15832<br>QQH15844
117.	4302	T	T	T	I<br>I	Nsp10	QQH16468<br>QQH16456
118.	4315	N	N	N	*	Nsp10	QQH16516
119.	4452	D	D	D	N	Nsp12	QQH16000
120.	4489	A	A	A	V<br>V<br>V<br>V<br>V<br>V<br>V<br>V<br>V<br><br>*<br>*<br>*<br>V<br>V<br>V<br>V	Nsp12	QQH16252<br>QQH17080<br>QQH16024<br>QQH16864<br>QQH16912<br>QQH17068<br>QQH17212<br>QQH17224<br>QQH15928<br><br>QQH15736<br>QQH16936<br>QQH16948<br>QLD99985<br>QPB18038<br>QQK53970<br>QQL14037
121.	4588	M	M	M	I	Nsp12	QQH16348
122.	4668	T	T	T	A<br>A	Nsp12	QQH17212<br>QQH17224
123.	4681	Y	Y	Y	*	Nsp12	QQH16204
124.	4683	D	D	D	*	Nsp12	QQH16144
125.	4684	Q	Q	Q	*	Nsp12	QQH16144
126.	4715	P	P	P	L<br>L<br>L<br>L<br>L<br>L<br>L<br>L	Nsp12	QQH16144<br>QQH16456<br>QQH16468<br>QQH16408<br>QQH16420<br>QQH17356<br>QQH16492<br>QQH16720

					L		QQH16276
					L		QQH16000
					L		QQH16744
					L		QQH16756
					L		QQH16444
					L		QQH15748
					L		QQH17104
					L		QQH17128
					L		QQH17164
					L		QQH16240
					L		QQH16204
					L		QQH17296
					L		QQH17044
					L		QQH16216
					L		QQH16120
					L		QQH16084
					L		QQH16132
					L		QQH16168
					L		QQH16180
					L		QQH16108
					L		QQH16156
					L		QQH16192
					L		QQH15664
					L		QQH15700
					L		QQH17176
					L		QQH16924
					L		QQH16804
					L		QQH15916
					L		QQH16060
					L		QQH17428
					L		QQH16888
					L		QQH16996
					L		QQH17452
					L		QQH16096
					L		QQH15988
					L		QQH15760
					L		QQH17152
					L		QQH17440
					L		QQH17308
					L		QQH17260
					L		QQH17032
					L		QQH16960
					L		QQH16792
					L		QQH16288
					L		QQH15964
					L		QQH17368
					L		QQH17380
					L		QQH17320
					L		QQH17332
					L		QQH17188
					L		QQH17116
					L		QQH17092
					L		QQH17056
					L		QQH17020
					L		QQH16840
					L		QQH16816

					L			QQH16768
					L			QQH16564
					L			QQH16552
					L			QQH16516
					L			QQH16396
					L			QQH16780
					L			QQH16384
					L			QQH16336
					L			QQH16312
					L			QQH16012
					L			QQH15676
					L			QQH15724
					L			QQH15772
					L			QQH15784
					L			QQH15820
					L			QQH15976
					L			QQH16528
					L			QQH16588
					L			QQH16636
					L			QQH16648
					L			QQH16660
					L			QQH16900
					L			QQH16972
					L			QQH16984
					L			QQH17140
					L			QQH17236
					L			QQH17248
					L			QQH17284
					L			QQH15688
					L			QQH17404
					L			QQH17416
					L			QQH17272
					L			QQH16708
					L			QQH16672
					L			QQH16372
					L			QQH15868
					L			QQH15904
					L			QQH16072
					L			QQH16360
					L			QQH16540
					L			QQH16684
					L			QQH16732
					L			QQH17200
					L			QQH17344
					L			QQH15952
					L			QQH16036
					L			QQH16048
					L			QQH17392
					L			QQH17212
					L			QQH17224
					L			QQH15928
					L			QQH15808
					L			QQH16876
					L			QQH16828
					L			QQH16696
					L			QQH16576

					L<br>L<br>L<br>L<br>L<br>L<br>L<br>L<br>L<br>L<br>L<br>L<br>L<br>L<br>L<br>L<br>L<br>L<br>L<br>L<br>L<br>L<br>L<br>L<br>L<br>L<br>L<br>L<br>L<br>L<br>L<br>L<br>L<br>L<br>L<br>L<br>L<br>L<br>L<br>L<br>L<br>L<br>L<br>L<br>L<br>L<br>L<br>L<br>L<br>L<br>L<br>L<br>L<br>L<br>L		QQH16504<br>QQH16480<br>QQH16600<br>QQH16432<br>QQH16348<br>QQH16348<br>QQH16612<br>QQH15832<br>QQH15844<br>QQH15892<br>QQH15940<br>QQH16264<br>QQH16300<br>QQH16324<br>QQH16852<br>QQH17008<br>QQH15796<br>QLD99687<br>QQL14073<br>QQL14166<br>QLD99663<br>QNC49338<br>QQL14178<br>QNV71166<br>QNV71178<br>QNV71190<br>QNV71202<br>QQL14274<br>QQL14025<br>QQL13896<br>QQL13931<br>QQL13954<br>QQL13989<br>QQK53886<br>QQL14013<br>QQL14001<br>QQL13908<br>QQL13872<br>QQK53898<br>QQK33666<br>QQS74323<br>QLD99699<br>QQD86525<br>QQL14049<br>QNV71214<br>EPI_ISL_1385796<br>EPI_ISL_1385798<br>EPI_ISL_1406395<br>EPI_ISL_1406397<br>EPI_ISL_1406400<br>EPI_ISL_468160<br>EPI_ISL_468161<br>EPI_ISL_468162<br>EPI_ISL_708839<br>EPI_ISL_708840
127.	4774	A	A	A	V	Nsp12	QQK53886

128.	4921	A	A	A	V	Nsp12	QQL14025
129.	4979	V	V	V	L	Nsp12	QQL13896
130.	4985	K	K	K	E	Nsp12	QQH16204
131.	5005			H	Y	Nsp12	EPI_ISL_1406397
132.	5006	L	L	L	F	Nsp12	QQH16144
133.	5031	A	A	A	V	Nsp12	QQH16840
134.	5222	P	P	P	S<br>S	Nsp12	QQH16420<br>QQH16408
135.	5305	S	S	S	L	Nsp12	QQH16960
136.	5425	D	D	D	N	Nsp13	QQH17308
137.	5451	T	T	T	I<br>I	Nsp13	QQL14073<br>QQL14166
138.	5533	V	V	V	F	Nsp13	QQK53886
139.	5561	A	A	A	T	Nsp13	QNC49338
140.	5606	G	G	G	V	Nsp13	QQH16216
141.	5614	H	H	H	Y	Nsp13	QQH16036
142.	5618	G	G	G	V	Nsp13	QQH17260
143.	5672	V	V	V	L	Nsp13	QQH16768
144.	5762	L	L	L	*	Nsp13	QQH17296
145.	5845	V	V	V	I	Nsp13	QQH17356
146.	5903	R	R	R	K	Nsp13	QQH16252
147.	5923	T	T	T	I<br>I	Nsp13	QQH17356<br>QQH15808
148.	5947	Q	Q	Q	H<br>H	Nsp14	QQH15808<br>QQH17356
149.	5951	H	H	H	Y<br>Y	Nsp14	QQH16504<br>QQH16000
150.	5961	E	E	E	*	Nsp14	QQH16144
151.	5969	G	G	G	*	Nsp14	QQH16144
152.	6044	A	A	A	S<br>S	Nsp14	QQH16420<br>QQH16408
153.	6067	P	P	P	*	Nsp14	QQH16156
154.	6113	H	H	H	*<br>*<br>*<br>*<br>*<br>*<br>*<br>*	Nsp14	QQH16120<br>QQH16084<br>QQH16132<br>QQH16168<br>QQH16180<br>QQH16108<br>QQH16156<br>QQH16192
155.	6175			T	I	Nsp14	EPI_ISL_468162
156.	6205	L	L	L	*<br>*<br>*<br>*	Nsp14	QQH17128<br>QQH17164<br>QQH15664<br>QQH16828
157.	6216	D	D	D	Y	Nsp14	QQH17356
158.	6236	K	K	K	N	Nsp14	QQH16288
159.	6285	A	A	A	S	Nsp14	QQH17056
160.	6297	T	T	T	I	Nsp14	QQH17392
161.	6418	Q	L	L	*	Nsp14	QIS60274
162.	6419	Y	Y	Y	*	Nsp14	QIS60274
163.	6420	L	L	L	*	Nsp14	QIS60274

164.	6421	D	D	D	*<br>Y<br>Y	Nsp14	QIS60274<br>QQH16456<br>QQH16468
165.	6422	A	A	A	*	Nsp14	QIS60274
166.	6423	Y	Y	Y	*	Nsp14	QIS60274
167.	6424	N	N	N	*	Nsp14	QIS60274
168.	6543	E	D	D	Y	Nsp15	QQK53970
169.	6518	V	V	V	L	Nsp15	QQH15952
170.	6525	N	N	N	*	Nsp15	QQH17296
171.	6579	V	V	V	F<br>F	Nsp15	QQH17056<br>QQH16756
172.	6581	G	G	G	V	Nsp15	QQH16432
173.	6657	P	P	P	L	Nsp15	QQH17440
174.	6661	M	M	M	*	Nsp15	QQH16216
175.	6670	M	M	M	I<br>I	Nsp15	QQH16276<br>QQK53898
176.	6898	L	L	L	F	Nsp16	QQH16492
177.	6960	A	A	A	*	Nsp16	QQH16936
178.	7024			A	S	Nsp16	EPI_ISL_1406395
179.	7058	M	M	M	I	Nsp16	QQK53898

This table shows the amino acid substitutions occurred in the polyprotein pp1ab of SARS-CoV-2 strains circulating in Pakistan during the 1st pandemic wave (March 01, 2020, to June 30, 2020). The amino acid position numbers in the 2nd column are according to the coordinates of polyprotein pp1ab of SARS-CoV-2. The 6th column indicates the amino acid substitutions in SARS-CoV-2 sampled from Pakistani population in relation to the reference sequence for the Wuhan strain and closely related bat-CoVs. The 7th column shows the type of nonstructural protein where the particular substitution resides. Please note: the data presented in this table is derived from all available completely sequenced genomes of SARS-CoV-2 sampled from the Pakistani population during the first wave of the pandemic (March 01, 2020, to June 30, 2020).

Table S4. The amino acid substitutions in the macrodomains of SARS-CoV-2 sampled from Pakistani population during first pandemic wave (March 01, 2020, to June 30, 2020).

S.No.	Amino Acid position	Bat-SL-CoV (AVP78030.1)	Bat-CoV-RaTG13 (QHR63299.1)	SARS-CoV-2 (YP_009724389.1)	Pak Isolates	Accession No.
1.	1083	M	M	M	I	EPI_ISL_1406395
2.	1125	G	G	G	C	QQH15880
3.	1175	L	L	L	I	QQL13872
4.	1246	T	T	T	I	QQH16492<br>QQH16720<br>QQH17404<br>QQH17416<br>QQH17272<br>QQH16708<br>QQH16672<br>QQH16372<br>QQH15868<br>QQH15904<br>QQH16072<br>QQH16360<br>QQH16540<br>QQH16684<br>QQH16732<br>QQH17200<br>QQH17344<br>QQH15952<br>QNV71166<br>QNV71178<br>QNV71190<br>QNV71202
5.	1299	V	V	V	L	EPI_ISL_1385798<br>EPI_ISL_1406400
6.	1305	K	K	K	N	QNV71166<br>QNV71178<br>QNV71190<br>QNV71202
7.	1355	I	I	I	V	QQH17368<br>QQH17380
8.	1396	K	K	K	R	QQL14037
9.	1398	I	I	I	T	QQH17092
10.	1426	T	T	T	*	QQH16000

Macrodomains of SARS-CoV-2 sampled from Pakistan during 1st pandemic wave have experienced 10 substitutions. The amino acid positions given in 2nd column are according to the coordinates of polyprotein pp1ab. The 6th column indicates the amino acid substitutions in Pakistan's isolates in relation to the reference sequence for the Wuhan strain (YP_009724389.1) and closely related bat-CoVs.

Table S5. Estimation of physicochemical impact of amino acid replacements in the macrodomains.

S.No	Substitution Position	Domain Type	Radical/ Neutral	Impact on Protein Stability (ΔΔG)
1.	M265I	Macrodomain-1	Radical (+1)	Reduced Stability (-0.48)
2.	G307C	Macrodomain-1	Radical (-3)	Reduced Stability (-2.87)
3.	L357I	Macrodomain-1	Radical (+2)	Increased Stability (0.65)
4.	T428I	Macrodomain-2	Radical (-1)	Increased Stability (0.78)
5.	V481L	Macrodomain-2	Radical (+1)	Reduced Stability (-1.45)
6.	K487N	Macrodomain-2	Neutral (0)	Reduced Stability (-0.14)
7.	I537V	Macrodomain-3	Radical (+3)	Increased Stability (0.02)
8.	K578R	Macrodomain-3	Radical (+2)	Increased Stability (0.26)
9.	I580T	Macrodomain-3	Radical (-1)	Reduced Stability (-1.79)
10.	T608*	Macrodomain-3	-	-

In total ten amino acid replacements were detected in the macrodomains of SARS-CoV-2 sampled from Pakistani population during first pandemic wave (March 01, 2020, to June 30, 2020). The amino acid positions given in 2nd column are according to the coordinates of Nsp3 protein. Locations of substitutions with respect to macrodomains type is provided in 3rd column. The 4th column depicts the putative physicochemical impact of each replacement on protein/structure function, the number within brackets are the log odds associated with changing the amino acids. Positive numbers imply a preferred change, zero implies a neutral change and negative numbers imply an un-preferred change. The 5th column depicts the putative impact of each replacement on the stability of protein structure. The number within brackets depicts the protein stability free energy change (ΔΔG) upon single amino acid substitution. ΔΔG (kcal/mole) is calculated by using structure information. Positive values imply an increase in the stability of protein structure. Conversely, negative numbers imply a decrease in the stability of protein structure.

Table S6. Structural deviations in the backbone torsion angles of Macrodomain-1 and Macrodomain-2.				
**S.No**	**Mutations**	**3D superimposition of protein structures**	**Changes in the backbone torsion angles. (Φ °, Ψ °) residue numbers**	**Changes in secondary structure elements (SSEs)**
1.	M256I **(Macrodomain-1)**	(PDB entry: 6W02) **Reference** (EPI_ISL_1406395) **Mutant**	4, 9, 14, 86, 87, 88, 89,167, 168	10 (β-sheet formation instead of loop) 11 (β-sheet formation instead of loop) 12 (β-sheet formation instead of loop) 13 (β-sheet formation instead of loop) 20 (Loop formation instead of β-sheet) 39 (β-sheet formation instead of loop) 57 (Loop formation instead of α-helix) 96 (β-sheet formation instead of loop) 100 (Loop formation instead of α-helix) 101 (Loop formation instead of α-helix) 102 (Loop formation instead of α-helix) 104 (Loop formation instead of α-helix) 105 (Loop formation instead of α-helix) 155 (Loop formation instead of β-sheet)
2.	G307C **(Macrodomain-1)**	(PDB entry: 6W02) **Reference** (QQH15880) **Mutant**	5, 14, 87, 88, 128, 129, 168	10 (β-sheet formation instead of loop) 11 (β-sheet formation instead of loop) 12 (β-sheet formation instead of loop) 13 (β-sheet formation instead of loop) 39 (β-sheet formation instead of loop) 57 (Loop formation instead of α-helix) 96 (β-sheet formation instead of loop)

3.	L357I<br>**(Macrodomain-1)**	(PDB entry: 6W02)<br>**Reference**<br><br>(QQL13872)<br>**Mutant**	4, 9, 14, 15, 47, 48, 119, 120, 130, 131	10<br>(β-sheet formation instead of loop)<br>11<br>(β-sheet formation instead of loop)<br>12<br>(β-sheet formation instead of loop)<br>13<br>(β-sheet formation instead of loop)<br>39<br>(β-sheet formation instead of loop)<br>57<br>(Loop formation instead of α-helix)<br>96<br>(β-sheet formation instead of loop)<br>100<br>(Loop formation instead of α-helix)<br>101<br>(Loop formation instead of α-helix)<br>102<br>(Loop formation instead of α-helix)<br>168<br>(α-helixformation instead of loop)
4.	T428I<br>**(Macrodomain-2)**	(YP_009725299.1)<br>**Reference**<br><br>(QQH16492)<br>**Mutant**	11, 24, 33, 34, 36, 39, 45, 46, 57, 99, 100, 136, 138	23<br>(α-helix formation instead of loop)<br>32<br>(α -helix formation instead of loop)
5.	V481L<br>**(Macrodomain-2)**	(YP_009725299.1)<br>**Reference**<br><br>(EPI_ISL_1385798)<br>**Mutant**	40, 41, 45, 46, 47, 99, 100, 135, 136, 137	32<br>(α -helix formation instead of loop)<br>87<br>(α -helix formation instead of loop)<br>109<br>(Loop formation instead of α-helix)<br>110<br>(Loop formation instead of α-helix)<br>111<br>(Loop formation instead of α-helix)
6.	K487N<br>**(Macrodomain-2)**	(YP_009725299.1)<br>**Reference**<br><br>(QNV71166)<br>**Mutant**	11, 24, 26, 33, 34, 35, 38, 44, 45, 136, 137	109<br>(Loop formation instead of α-helix)<br>110<br>(Loop formation instead of α-helix)<br>111<br>(Loop formation instead of α-helix)

This table shows the effect of amino acid substitutions on the backbone torsion angles and secondary structure elements of Mac-1 and Mac-2 of SARS-CoV-2 sampled from Pakistan during first pandemic wave (March 01, 2020, to June 30, 2020). Protein structural effects were estimated in relation to the reference sequences/structures (6W02 for Mac-1 and YP_009724389.1 for Mac2).

Table S7. ADPr-binding analysis of macrodomain-1.

S.No	Virus Type	Interacting residue with ADPr	Docking Score (kcal/mol)
1.	SARS-CoV-2 (PDB entry: 6W02) **Reference Mac-1**	Asp22, Ile23, Asn40, Lys44, Gly46, Gly48, Val49, Ser128, Ala129, Ile131, Phe132, Phe156	-9.9
2.	SARS-CoV-2 (EPI_ISL_1406395) **Mac-1 mutant (M265I)**	Ala38, Leu126, Gly130, Ile131, Phe132, Ala154, Asp157	-7.3
3.	SARS-CoV-2 (QQH15880) **Mac-1 mutant (G307C)**	Asp22, Gly46, Gly48, Leu126, Ser128, Ala129, Asp157	-6.6
4.	SARS-CoV-2 (QQL13872) **Mac-1 mutant (L357I)**	Gly46, Leu126, Ala154, Asp157, Phe156	-6.7

This table summarizes the results of molecular docking of ADP-ribose molecule with the macrodomain-1 of SARS-CoV-2. 2nd column: this analysis involves reference Mac-1 structure (PDB entry: 6W02) and three mutant versions of Mac-1 identified in the present study (M265I, G307C and L357I). The 3rd column shows the amino acid residues of macrodomain-1 which participate in the hydrogen bonding with the ADP-ribose molecule. Docking scores (kcal/mol) are given in the 4th column. The lowest the docking score the higher is the binding affinity. ADPr; adenosine diphosphate (ADP-ribose).

**Table S8: Molecular Mechanics/Generalized Born Surface Area (MM/GBSA) based free energy calculations.**					
**Complex name**	**vdW**	**Electrostatic energy**	**GB**	**ESURF**	**Total binding energy**
**Wild type Mac1-ADPr**	-51.4594	-147.4858	168.6718	-5.8393	-36.1127
**Mutant Mac1(M265I)-ADPr**	-41.3404	-90.9679	114.7636	-4.8410	-22.3857
**Mutant Mac1(G307C)-ADPr**	-33.3470	-50.8977	70.0676	-3.7984	-17.9755
**Mutant Mac1(L357I)-ADPr**	-26.3813	-51.0053	65.1276	-3.3125	-15.5715

This table illustrates the Molecular Mechanics/Generalized Born Surface Area (MM/GBSA) based free binding energy calculations of wild-type and mutant Mac1(M265I, G307C, L357I)-ADPr complexes. The column 2nd . 3rd, 4th and 5th represent the Vander Waal energy, electrostatic energy, polar solvated energy by Generalized born model and nonpolar solvation energy, respectively. The 6th column represents the total binding energy of all complexes. All energies are calculated in kcal/mol.

Table S9: The non-structural protein-3 sequences derived from the genomes of subsequent variants of SARS-CoV-2 emerged after the first pandemic wave from Pakistan, spanning July 1,2020 to February 2024.

S.No	Protein accession	Variant	Collection data	Locality
1.	EPI_ISL_15172250	Alpha	2021-03-26	Pakistan
2.	EPI_ISL_15172251	Alpha	2021-03-26	Pakistan
3.	EPI_ISL_15172252	Alpha	2021-03-26	Pakistan
4.	EPI_ISL_15172254	Alpha	2021-03-26	Pakistan
5.	EPI_ISL_15172256	Alpha	2021-03-26	Pakistan
6.	EPI_ISL_15172257	Alpha	2021-03-26	Pakistan
7.	EPI_ISL_15172259	Alpha	2021-03-26	Pakistan
8.	EPI_ISL_18056939	Alpha	2021-07-05	Pakistan
9.	EPI_ISL_18056940	Alpha	2021-07-05	Pakistan
10.	EPI_ISL_18056941	Alpha	2021-07-05	Pakistan
11.	EPI_ISL_11793997	Beta	2021-07-04	Pakistan
12.	EPI_ISL_11793998	Beta	2021-07-05	Pakistan
13.	EPI_ISL_16925128	Beta	2021-06-24	Pakistan
14.	EPI_ISL_17814257	Beta	2021-05-29	Pakistan
15.	EPI_ISL_18056943	Beta	2021-06-27	Pakistan
16.	EPI_ISL_18056945	Beta	2021-05-16	Pakistan
17.	EPI_ISL_18056956	Beta	2021-07-01	Pakistan
18.	EPI_ISL_18242884	Beta	2021-07-01	Pakistan
19.	EPI_ISL_4458435	Beta	2021-05-17	Pakistan
20.	EPI_ISL_8317245	Beta	2021-06-01	Pakistan
21.	EPI_ISL_18123260	Delta	2021-08-17	Pakistan
22	EPI_ISL_18123261	Delta	2021-08-19	Pakistan
23	EPI_ISL_18123265	Delta	2021-08-10	Pakistan
24	EPI_ISL_18134828	Delta	2022-02-02	Pakistan
25	EPI_ISL_18134833	Delta	2021-07-17	Pakistan
26	EPI_ISL_18134834	Delta	2021-07-17	Pakistan
27	EPI_ISL_18134836	Delta	2021-07-17	Pakistan
28	EPI_ISL_18134837	Delta	2021-07-17	Pakistan
29	EPI_ISL_18254343	Delta	2021-07-05	Pakistan
30	EPI_ISL_18254351	Delta	2021-06-28	Pakistan
31	EPI_ISL_15492851	Omicron	2022-06-29	Pakistan
32	EPI_ISL_15840454	Omicron	2022-06-27	Pakistan
33	EPI_ISL_17236580	Omicron	2022-01-29	Pakistan
34	EPI_ISL_17425634	Omicron	2022-03-09	Pakistan
35	EPI_ISL_17560618	Omicron	2023-03-31	Pakistan
36	EPI_ISL_17560619	Omicron	2023-04-05	Pakistan
37	EPI_ISL_17560621	Omicron	2023-04-06	Pakistan
38	EPI_ISL_17560622	Omicron	2023-04-12	Pakistan
39	EPI_ISL_18123271	Omicron	2021-12-21	Pakistan
40	EPI_ISL_18445462	Omicron	2023-08-30	Pakistan

This table provides the accession numbers of Non-structural protein 3 (nsp3), variant name, sample collection date, and source locality. Please note; the data presented in this table is obtained from GISAID belongs genomes of SARS-CoV-2 that sampled after first pandemic wave from Pakistan (July 01, 2020 till date).

**Table S10: the amino acid substitutions in the macrodomains of SARS-CoV-2 variant that emerged after first pandemic wave in Pakistan, spanning July 1,2020 to February 2024.**			
**S.No**	**Mutation**	**Macrodomains**	**Accession ID**
1.	T217I	Mac-1	Beta/EPI_ISL_18056945<br>Beta/EPI_ISL_18056956
2.	D309N	Mac-1	Delta/EPI_ISL_18123261
3.	P402S	Mac-2	Alpha/EPI_ISL_15172252
4.	K412N	Mac-2	Alpha/EPI_ISL_15172254
5.	A488S	Mac-2	Delta/EPI_ISL_18123260<br>Delta/EPI_ISL_18123261<br>Delta/EPI_ISL_18123265<br>Delta/EPI_ISL_18134828<br>Delta/EPI_ISL_18134833<br>Delta/EPI_ISL_18134834<br>Delta/EPI_ISL_18134836<br>Delta/EPI_ISL_18134837<br>Delta/EPI_ISL_18254343<br>Delta/EPI_ISL_18254351
6.	G489S	Mac-2	Omicron/EPI_ISL_15492851<br>Omicron/EPI_ISL_15840454<br>Omicron/EPI_ISL_17236580<br>Omicron/EPI_ISL_17425634<br>Omicron/EPI_ISL_17560618<br>Omicron/EPI_ISL_17560619<br>Omicron/EPI_ISL_17560621<br>Omicron/EPI_ISL_17560622<br>Omicron/EPI_ISL_18123271<br>Omicron/EPI_ISL_18445462
7.	T504A	Mac-2	Alpha/EPI_ISL_18056940
8.	V613V	Mac-3	Beta/EPI_ISL_18056943
9.	H682H	Mac-3	Alpha/EPI_ISL_18056940
This table indicates the nine amino substitutions in the macrodomains of SARS-CoV-2 variants that emerged after first pandemic wave. The amino acid positions given in 2nd column are according to the coordinates of Nsp3 protein. Locations of substitutions with respect to macrodomains type is provided in 3rd column. The 4rth column indicate the accession IDs of sequences in which corresponding mutation reside.			
